# Tumor microenvironment–responsive and modulatory manganese-based nanoenzyme for enhanced tumor immunotherapy

**DOI:** 10.3389/fphar.2024.1518983

**Published:** 2025-01-03

**Authors:** Qi Yang, Qiong Wu, Haiyan Liu, Jiandong Wu, Feng Ma, Xiaofeng Tian

**Affiliations:** ^1^ Department of Gynecology and Obstetrics, China-Japan Union Hospital of Jilin University, Changchun, Jilin, China; ^2^ Key Laboratory of Pathobiology, Ministry of Education, Nanomedicine and Translational Research Center, China-Japan Union Hospital of Jilin University, Changchun, Jilin, China; ^3^ Key Laboratory of Pathobiology Ministry of Education, Department of Anatomy, College of Basic Medical Sciences, Jilin University, Changchun, China; ^4^ Department of Pathology, China-Japan Union Hospital of Jilin University, Changchun, Jilin, China; ^5^ Department of General Surgery, China-Japan Union Hospital of Jilin University, Changchun, Jilin, China

**Keywords:** nanoenzyme, tumor microenvironment, catalytic immunotherapy, manganese, polydopamine

## Abstract

The characteristics of the tumor microenvironment (TME) have a close and internal correlation with the effect of cancer immunotherapy, significantly affecting the progression and metastasis of cancer. The rational design of nanoenzymes that possess the ability to respond to and regulate the TME is driving a new direction in catalytic immunotherapy. In this study, we designed a multifunctional manganese (Mn)-based nanoenzyme that is responsive to acidic pH and overxpressed H_2_O_2_ at tumor site and holds capability of modulating hypoxic and immunosuppressive TME for synergistic anti-tumor photothermal/photodynamic/immunotherapy. We found that this artificial nanoenzyme promoted peroxidase-like and catalase-like activities and catalyzed the *in-situ* decomposition of H_2_O_2_, a metabolic waste product in the TME, into ∙OH and O_2_, resulting in a ROS burst for killing tumors and relieving hypoxic TME to enhance cancer therapy. Besides the photothermal effect and the enhancement of ROS burst-induced immunogenic cell death, combination of Mn^2+^ released from Mn-based nanoenzyme in acidic TME and programmed death-ligand 1 blockade triggered a significant anti-tumor immune response. A remarkable *in vivo* synergistic therapeutic effect was achieved with effective inhibition of primary tumor growth and lung metastasis. Therefore, this TME-responsive Mn-based nanoenzyme offers a safe and efficient platform for reversing the immunosuppressive microenvironment and achieving synergistic anti-tumor immunotherapy.

## 1 Introduction

Nanoenzymes are nanomaterials with catalytic activity similar to that of natural enzymes, capable of catalysing various biochemical reactions under physiological or specific environmental conditions. They offer advantages such as stable activity, lower susceptibility to temperature and pH changes, easy accessibility, and multifunctionality. In recent years, the application of nanoenzymes has evolved from *in vitro* diagnosis to *in vivo* catalytic therapy (Wu et al., 2024). For example, diversiform nanoenzymes have been developed to selectively target and kill tumor cells via catalytic conversion of the tumor metabolites into reactive oxygen species (ROS) in response to specific physiological stimuli, thereby inducing localized oxidative stress and apoptosis. The rapid development of “nano-catalytic medicine” offers a novel therapeutic approach for recalcitrant tumors (Li M. Y. et al., 2024; Wen et al., 2019; Yu et al., 2022).

The tumor microenvironment (TME), which includes malignant and non-malignant cells, blood vessels, extracellular matrix, and various secreted factors, is characterized by hypoxia, acidity, overexpressed hydrogen peroxide (H_2_O_2_), high levels of ROS, and an immunosuppressive microenvironment (Pitt et al., 2016). These conditions create a challenging environment for conventional therapeutic agents, thereby limiting their efficacy (Nakamura and Smyth, 2020). To address this, TME-responsive nanoenzymes are currently being developed to selectively accumulate at the tumor site and realize tumor-specific cancer treatment (Xiao and Yu, 2021). Cancer therapies that achieve modulation of the TME inside solid tumors have been demonstrated to promote the therapeutic outcomes (Cowman and Koh, 2022). Several types of nanoenzymes have been designed to alleviate hypoxia, overcome stromal barriers, and modulate immunosuppressive conditions, thereby improving immune cell infiltration and activation of both systemic and local immune responses (Yang et al., 2023). Challenges facing nanoenzymatic catalysed immunisation at this stage include low nanoenzymatic activity, poor catalytic specificity, limited types of enzymes, enzyme recyclability and material toxicity (Tian et al., 2024). Encouragingly, there has been a great deal of research on silica-based nanoenzymes and metal-based nanoenzymes that have provided us with new ideas in cancer immunotherapy and clinical translational challenges, but further in-depth studies are still needed (Theivendran et al., 2023; Luo Y. et al., 2024). In order to design effective therapeutic nanoenzymes, we need to understand the properties of the nanoenzymatic material, the biological system of the tumour, the tumour microenvironment and their interactions (Zhang et al., 2024). Hence, fabricating TME-responsive and modulating nanoenzymes that can realize targeted delivery and synergistic therapeutic outcomes, and rebuild a favorable TME for anti-tumor activity may be of great significance for enhancing nanocatalysis therapeutic efficacy and extending the current nanoenzyme-therapeutic armamentarium.

In recent years, MnO_2_ nanostructures as theranostic agents have attracted wide attention owing to its TME responsiveness and multiple enzymatic activities (Chen et al., 2022). Within the TME, MnO_2_ nanostructures would slowily disintegrate and are convert into to harmless water-soluble Mn^2+^ ions by reacting with either H^+^ or high-level glutathione (GSH) (Lu et al., 2022; Tian et al., 2019; Yue et al., 2024). Manganese is critical for antitumor immune responses via cGAS-STING (He et al., 2023) and promote CD8^+^ T cell activation, and the combination of Mn^2+^ and PD-1 antibody can significantly enhance the tumor treatment effect of PD-1 antibody (Sun et al., 2023), improving the efficacy of clinical immunotherapy.

**Scheme 1:**
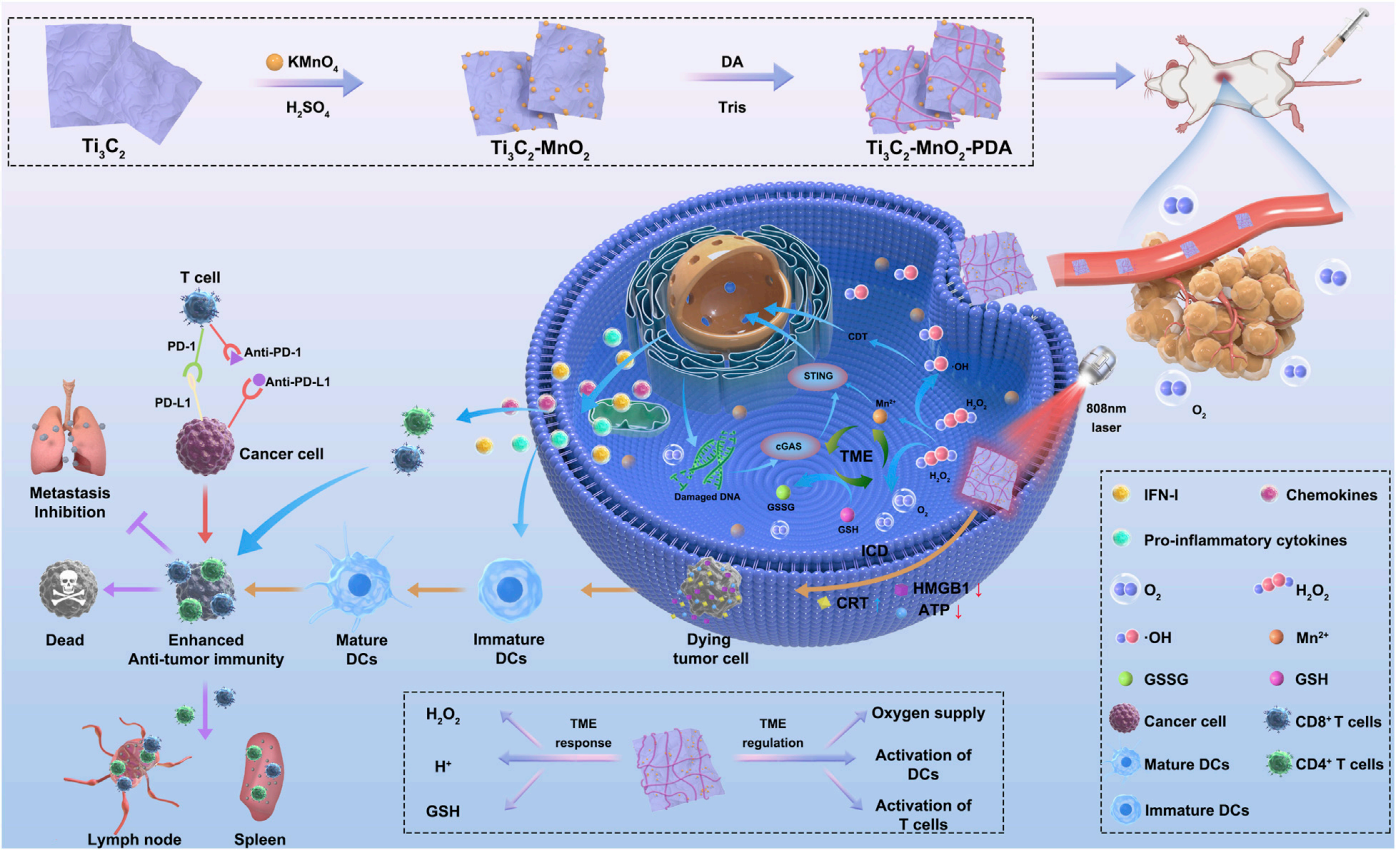
Schematic illustration of TME-responsive and TME-regulated nanoenzymes (Ti_3_C_2_-MnO_2_-PDA). (1) Preparation of Ti_3_C_2_-MnO_2_-PDA. (2) The nanoenzyme breaks down H_2_O_2_ into ∙OH and O_2_ in the acidic TME. MnO_2_ consumes the high levels of GSH in the tumor site to generate GSSG and Mn^2+^. (3) Activation of STING and cGAS signaling pathways by Mn^2+^ and PTT-induced ICD activate DCs, which in combination with anti-PD-L1 antibodies synergistically trigger local and systemic T cell responses against tumor growth and metastasis. Abbreviations: Ti_3_C_2_, titanium carbide; MnO_2_, manganese dioxide; PDA, polydopamine; H_2_O_2_, hydrogen peroxide; TME, tumor microenvironment; GSH, glutathione; GSSG, glutathione disulfide; STING, stimulator of interferon genes; cGAS, cyclic guanosine monophosphate–adenosine monophosphate synthase; PTT, photothermal therapy; ICD, immunogenic cell death; DC, dendritic cell; PD-L1, programmed death-ligand 1; IFN-1, interferon type I.

In this study, we developed a novel manganese (Mn)-based nanoenzyme using MnO_2_ loaded titanium carbide (Ti_3_C_2_) nanosheets with high photothermal conversion, and this nanocomposite was further coated with polydopamine (PDA) film for achieving better stability and biocompatibility (Scheme 1). The proposed Ti_3_C_2_-MnO_2_-PDA was acid-responsive since PDA could slowly disintegrate in acidic conditions at tumor sites and expose MnO_2_, meanwhile, this nanocomposite exhibited peroxidase-like activity and catalase-like activity which can catalyze the production of toxic ∙OH radicals from H_2_O_2_ to enable the catalytic therapy with local oxygen supply (Lin et al., 2018b; Ma et al., 2021). Due to the high photothermal conversion efficiency (η = 55%) of Ti_3_C_2_-MnO_2_-PDA under 808 nm irradiation, the nanoenzymes also enabled PTT that combined catalysis for inducing ROS burst, improving therapeutic efficiency (Sun J. et al., 2022; Sun P. et al., 2022). Furthermore, the MnO_2_ shell disintegrated in the presence of high levels of GSH (Lin et al., 2018a; Li W. et al., 2024) and acidic pH, and Mn^2+^ released from the nanocomposite acted as an agonist of the cGAS-STING pathway and directly activates STING to induce secretion of type I interferons, pro-inflammatory cytokines, and chemokines, facilitating CD8^+^ T cell activation (Lu et al., 2022; Lv et al., 2020; Sun et al., 2021; Wang et al., 2021; Zhao et al., 2023) synergy of ROS burst-induced immunogenic cell death (ICD) and Mn^2+^/PD-Ll antibody immunotherapy triggered a significant anti-tumor immune response, leading to a remarkable inhibition of primary tumor growth and lung metastasis (Cheng et al., 2022).

## 2 Materials and methods

### 2.1 Reagents

Ti_3_AlC_2_ was purchased from Jilin Yiyi Technology Co. (Changchun, China) and potassium permanganate (KMnO_4_) from Xintong Fine Chemical Co. Ltd. (Tianjin, China). RPMI 1640 medium was purchased from Gibco (Grand Island, NY, United States), fetal bovine serum from Biological Industries (Belt Haemek, Israel), trypsin-EDTA from NCM Biotechnology (Suzhou, China), and penicillin-streptomycin from Cytiva (Beijing, China). Mounting medium with DAPI-(Aqueous) and fluoroshield solution were purchased from Abcam (Lot: GR3369395-5; Cambridge, United Kingdom). Ti_3_C_2_-MXene was purchased from JiLin Yiyi Technology Co. Ltd. (Changchun, China), 3,3′,5,5′-tetramethylbenzidine (TMB) from Beyotime Biotechnology (Shanghai, China), and H_2_O_2_ from Beijing Chemical Works (Beijing, China). InVivoMAb anti-mouse programmed death-ligand 1 (PD-L1) (B7-H1) (BE0101) was acquired from BioXCell (Lebanon, NH, United States). The water used in all experiments was prepared using a Millipore purification device. Cell counting kit-8 (CCK-8) was purchased from Bimake (Beijing, China). Rabbit anti-calreticulin (anti-CRT) and rabbit anti-high mobility group box 1 (anti-HMGB1) antibodies were purchased from Abcam. The ATP measurement kit and calcein-AM/PI kit were purchased from Beyotime Biotechnology. Collagenase, hyaluronidase, and DNase I were purchased from Yuanye Biotechnology (Shanghai, China). The antibodies used for flow cytometry are listed in Supplementary Table S2.

### 2.2 Preparation of Ti_3_C_2_-MnO_2_-PDA

To prepare Ti_3_C_2_-MnO_2_, 6 mL of Ti_3_C_2_-MXene (5 mg/mL) was added to 33 mL of purified water in a 50-mL conical flask and stirred. Thereafter, 6 mL of aqueous KMnO_4_ (50 mg/mL) and 150 µL of concentrated H_2_SO_4_ were added to the mixture, and then stirred for 30 min. The solution was then centrifuged, and the precipitated Ti_3_C_2_-MnO_2_ was resuspended in deionized water. To synthesize Ti_3_C_2_-MnO_2_-PDA, 4 mL of Ti_3_C_2_-MXene-MnO_2_ (5 mg/mL) was added to 26 mL of deionized water. Following sequential addition of 60 mg dopamine and 320 µL of Tris (1 M), the solution was stirred at 2,000 rpm for 6 h. The supernatant was removed by centrifugation at 16,000 rpm and resuspended in deionised water after sonication with an ultrasonicator.

### 2.3 Characterization of Ti_3_C_2_-MnO_2_-PDA

The morphology of the Ti_3_C_2_-MnO_2_-PDA particles was analyzed using a transmission electron microscope (TEM). Crystal structure analysis was conducted using a Bragg–Brentano diffractometer (D8-Tools; Crystal Impact, Bonn, Germany) equipped with a Cu Kα (λ = 0.15418 nm) emitting source. X-ray photoelectron spectroscopy (XPS) was conducted on an Escalab-250 instrument (Thermo Fisher Scientific, Waltham, MA, United States) equipped with a hemisphere detector and a monochromatic Al Kα radiation source (1,486.6 eV). The morphology analysis was performed using a scanning electron microscope (SEM) (JSM6700F; JEOL Ltd., Tokyo, Japan) and a TEM) (JEM-2000EX; JEOL Ltd.). The UV-Vis spectra were obtained using a spectrophotometer (Tecan Austria GmbH, Grödig, Austria). The zeta potential of Ti_3_C_2_-MnO_2_-PDA was determined using a Nano Zetasizer (Malvern, UK).

### 2.4 Photothermal conversion efficiency and photothermal stability analyses

Different concentrations of Ti_3_C_2_-MnO_2_-PDA (0, 20, 40, 60, and 80 μg/mL) were irradiated with an 808 nm laser at 1 W/cm^2^ (Laserwave, LWIRPD-20F, Beijing, China). In addition, 40 μg/mL Ti_3_C_2_ -MnO_2_-PDA was irradiated with the laser at 1.5, 1, 0.8, and 0.6 W/cm^2^ for 5 min. The temperature changes were recorded using a thermal imager (FOTRIC,322Pro, Shanghai, China).

### 2.5 Cell culture

Mouse-derived 4T1 cells, human breast cancer cell (MDA-MB-231) and human breast normal epithelial cell (MCF-10A) were (Pricella,Wuhan, China) cultured at 37°C in 5% CO_2_, using 1,640 media with 10% FBS and 1% penicillin-streptomycin.

### 2.6 Electron microscopy

The 4T1 cells were cultured for 24 h at 37°C with 5% CO_2_, and then incubated with or without 40 μg/mL Ti_3_C_2_-MnO_2_-PDA for 24 h. The cells were fixed with pre-cooled 0.5 mL glutaric acid for 4–6 h, sliced, and viewed under an electron microscope.

### 2.7 CCK-8 assay

MCF-10A cells were seeded into 96-well plates at a density of 1 × 10^4^ cells per well and cultured overnight. Following incubation with different concentrations of Ti_3_C_2_-MnO_2_-PDA (0, 2.5, 5, 10, 20, 40, 60, and 80 μg/mL) for 24 h, CCK-8 reagent was added to each well. After incubating for 1 h, the absorbance of the cells was measured at 450 nm, and the viability was calculated. In another experiment, 4T1 cells (1 × 10^4^ cells per well) and MDA-MB-231 cells (1 × 10^4^ cells per well) were passaged in 96-well plates overnight, and incubated with different concentrations of Ti_3_C_2_-MnO_2_-PDA (0, 5, 10, 20, 40, 60, and 80 μg/mL) for 4 h. The cells were then irradiated with an 808 nm laser (1 W/cm^2^) for 5 min, and then incubated for 20 h. The CCK-8 assay was performed as described above.

### 2.8 Immunofluorescence assay

Immunogenic cell death (ICD) was evaluated *in vitro* by measuring CRT and HMGB1 expression using immunofluorescence. Briefly, 4T1 cells were seeded on coverslips placed in 24-well plates at a density of 2 × 10^4^ cells per well, and then cultured for 24 h. The cells were incubated with phosphate-buffered saline (PBS) or Ti_3_C_2_-MnO_2_-PDA for 6 h, and then irradiated with an 808 nm near infra-red (NIR) laser. Respective non-irradiated controls were also included. After washing three times with cold PBS, the cells were fixed with 300 μL of 4% paraformaldehyde for 30 min at 23°C–25°C. The cells were washed once with cold PBS, and permeabilized with 0.1% TritonX-100 in PBS for 30 min at room temperature. The cells were blocked with 1% BSA (Yuanye, Shanghai,China) at room temperature for 1 h, incubated overnight with the primary antibody at 4°C, and washed three times with PBST. Following incubation with Alexa Fluor 488-labeled secondary antibody at room temperature for 1 h, the coverslips were sealed with DAPI-containing glue, and viewed under a confocal microscope (Olympus).

### 2.9 ATP measurement assay

The 4T1 cells were seeded in 24-well plates at a density of 5 × 10^4^ cells per well, and then cultured for 24 h. ATP levels in the supernatants were measured using an enzyme-linked immunosorbent assay (luminescence) kit according to the manufacturer’s instructions.

### 2.10 Peroxidase reaction assay

To measure the peroxidase activity of Ti_3_C_2_-MnO_2_-PDA *in vitro*, the nanoenzymes (80 μg/mL) were incubated separately with 0.4 mM TMB buffer (pH 6.0), 50 μM H_2_O_2_, and a combination of both TMB and H_2_O_2_. As a control, TMB and H_2_O_2_ were incubated in the absence of the nanoenzyme. The absorbance at 652 nm was measured using a UV spectrophotometer (Tecan).

In addition, the peroxidase-like activity of the nano-enzymes was investigated by the catalytic oxidation reaction of TMB at different pH of 37°C. And the kinetic performance of the catalytic reaction of the nano-enzymes was observed by fixing the concentration of TMB and varying the concentration of H_2_O_2_ and fixing the concentration of H_2_O_2_ and varying the concentration of TMB, and the actual reaction rate was calculated by the amount of change in absorbance per unit time of the reaction system at 652 nm, and the values of Vmax and Km were calculated based on the Michaelis-Menten equation.

### 2.11 In vitro ∙OH generation

2 mL of mixture containing H_2_O_2_ (50 mM), MB (10 *μ*g/mL) was incubated with various concentrations of Ti_3_C_2_-MnO_2_-PDA (0, 1.25, 2.5, 5, 10, 20, 40 *μ*g/mL) (pH = 6) for 24 h. 2 mL of mixture containing H_2_O_2_ (50 mM), MB (10 *μ*g/mL) and Ti_3_C_2_-MnO_2_-PDA (40 *μ*g/mL) (pH = 5, 6, 7.4). After centrifugation, the supernatant was collected and the absorbance was recorded by UV–vis spectrophotometer. The absorbance changes at 670 nm were also recorded.

### 2.12 ROS assay

ROS generation was measured using a 2′,7′-dichlorodihydrofluorescein diacetate (DCFH-DA) fluorescence probe. Briefly, 4T1 cells were seeded into 24-well plates loaded with polylysine-coated crawler sheets (WHB) at a density of 5 × 10^4^ cells per well. Following overnight culture, the cells were incubated with 60 μg/mL Ti_3_C_2_-MnO_2_-PDA for 4 h. DCFH-DA (10 μM) was then added to each well, and the cells were incubated for an additional 30 min. The cells were washed twice with PBS and observed under a laser scanning confocal microscope (FV1000; OLYMPUS, Tokyo, Japan).

### 2.13 Cell death and viability assay

The 4T1 cells were seeded into 24-well plates loaded with polyline-coated crawler sheets at a density of 5 × 10^4^ cells per well and cultured for 24 h. The following day, the cells were incubated with PBS or 60 μg/mL Ti_3_C_2_-MnO_2_-PDA for 4 h, and then irradiated with an 808 nm laser (1 W/cm^2^) for 5 min. Non-irradiated controls were also included. The treated cells were then subsequently incubated for 20 h, harvested, and stained using a calcein-AM/PI kit according to the manufacturer’s instructions (Beyotime Biotechnology) to determine viability.

### 2.14 Apoptosis assay

The 4T1 cells were seeded into 24-well plates at a density of 5 × 10^4^ cells per well and cultured for 24 h. After incubating with PBS or Ti_3_C_2_-MnO_2_-PDA for 6 h, the cells were irradiated with an 808 nm NIR laser (1.5 W/cm^2^) for 5 min. Non-irradiated controls were also included. The cells were further incubated for 24 h and digested using EDTA-free trypsin (Biosharp). The harvested cells were centrifuged and resuspended in 200 µL of binding buffer, and then sequentially stained with 0.75 µL of annexin-V (Beyotime Biotechnology) for 10 min and 4 µL of 7AAD for 5 min in the dark. The samples were analyzed using flow cytometry (3 × 10^4^/sample,n = 4) to determine apoptosis rates.

### 2.15 Animal experiments

Six-to-eight-week old female BALB/c mice (weighing approximately 20 g) were purchased from Changchun Yisi Laboratory Animal Technology Co. (Changchun, China). The animal experiments were approved by the Institutional Animal Care and Use Committee of Jilin University (IACUC). The animal ethical clearance project number is SY202309038.

#### 2.15.1 *In vivo* pharmacokinetics and biodistribution of nanoenzymes

The 4T1 tumor-bearing BALB/C mice (n = 3) were intravenously injected with 10 mg/kg Cy5-labeled Ti_3_C_2_-MnO_2_-PDA in 100 μL of PBS once the tumors grew to approximately 300 mm^3^. Tumour volume is calculated on the basis of: Volume (V) = Length (L) × Width (W)^2^/2. Body weight and tumour volume were monitored throughout the experiment, and tumour diameters did not exceed 1.5 cm in the loaded experiments. Euthanasia was performed using inhaled carbon dioxide. The mice were euthanized at different time intervals after injection (1, 3, 6, 9, 12, and 24 h), and the tumors and major organs (heart, liver, spleen, lung, and kidney) were harvested for fluorescence imaging analysis (IVIS Spectrum; Perkin Elmer, Waltham, MA, United States).

#### 2.15.2 *In vivo* biosafety

The mice were intravenously injected with 100 μL of PBS or the same volume of 10 mg/kg Ti_3_C_2_-MnO_2_-PDA (n = 8). The general status of the mice was evaluated daily, and the body weight was measured on alternate days. All mice were euthanized on day 14 post-injection, and their blood samples and major organs, including heart, liver, spleen, lung, and kidney, were harvested. Systemic toxicity was evaluated by measuring the levels of alkaline phosphatase, alanine aminotransferase, aminotransferase, creatinine, and urea nitrogen in the blood samples (Nanjing Jianjian Bioengineering Institute, Nanjing, China). The organs were fixed in 4% neutral buffered formalin, embedded in paraffin, and cut into 5-μm-thick sections (CITOGLAS). The tissue sections were stained with hematoxylin and eosin (H&E) and take photos using ZEISS (Oberkochen, Germany).

The blood samples were centrifuged (3,000 rpm/min, 5 min), and the sedimented erythrocytes were washed three times with PBS and re-dispersed in 5 mL of PBS. Hemolysis assay was performed by incubating 200 μL of aliquots of the RBC suspension with deionized water (positive control), PBS (negative control), and different concentrations of Ti_3_C_2_-MnO_2_-PDA (5, 10, 20, 40, 60, and 80 μg/mL) at 37°C for 3 h. The samples were centrifuged at 1,000 *g* for 15 min, and the supernatants were dispensed into a 96-well microtiter plate. The absorbance (A) was measured at 570 nm, and hemolysis rate (%) was calculated as [A_sample_ - A_negative_/A_water_ - A_negative_] × 100%.

#### 2.15.3 *In vivo* imaging

To establish a tumor model, 5 × 10^5^ 4T1 cells were inoculated into the left mammary fat pad of each mouse. Tumor growth was regularly monitored, and the volume was calculated as (V) = Length (L) × Width (W)^2^/2. Once the tumors grew to approximately 100 mm^3^, the mice were injected into the tail vein with 10 mg/kg Ti_3_C_2_-MnO_2_-PDA in 100 μL of PBS. After laser irradiation (808 nm laser, 1 W/cm^2^), the *in vivo* thermal signals were detected at different time points (0, 1, 3, 5 min) using a thermal imager (FOTRIC, 322Pro, Shanghai, China) and the temperature of the thermal signals was measured.

#### 2.15.4 *In vivo* anti-tumor effect

The tumor-bearing mice were divided into the PBS, Ti_3_C_2_-MnO_2_-PDA (10 mg/kg), anti-PD-L1, Ti_3_C_2_-MnO_2_-PDA + anti-PD-L1, PBS + NIR,Ti_3_C_2_-MnO_2_-PDA + NIR, anti-PD-L1 + NIR, and Ti_3_C_2_-MnO_2_-PDA + anti-PD-L1 + NIR groups (n = 8). One h after injecting the different agents, the mice were irradiated with an 808 nm laser (1 W/cm^2^) for 5 min as appropriate. The temperature changes in the tumor were recorded in real-time using an infra-red thermal imager (322Pro; Fotric, Beijing, China). All mice were treated daily. The body weight and tumor volume were monitored every 2 d for 14 d. At the end of the treatment period, the mice were euthanized and the tumors were dissected. One part of the tumor specimens was fixed in 4% neutral buffered formalin, embedded in paraffin, and cut into 5-μm-thick sections for Ki-67 and TUNEL staining. The other part was processed for flow cytometry. (GB121141-100,G1501,Servicebio, Wuhan, China).

## 3 Statistical analysis

Data were presented as mean ± standard deviations (SDs) or standard error of mean. Statistical significance was calculated by t-test. Statistical analysis was carried out using GraphPad Prism 9.0 and Microsoft Excel 2020. Flowjo 10.0 was used for streaming data analysis. *p < 0.05; **p < 0.01; ***p < 0.001; ****p < 0.0001; ns stands for not significant.

## 4 Results and discussion

### 4.1 Preparation and characterization of Ti_3_C_2_-MnO_2_-PDA

**FIGURE 1:**
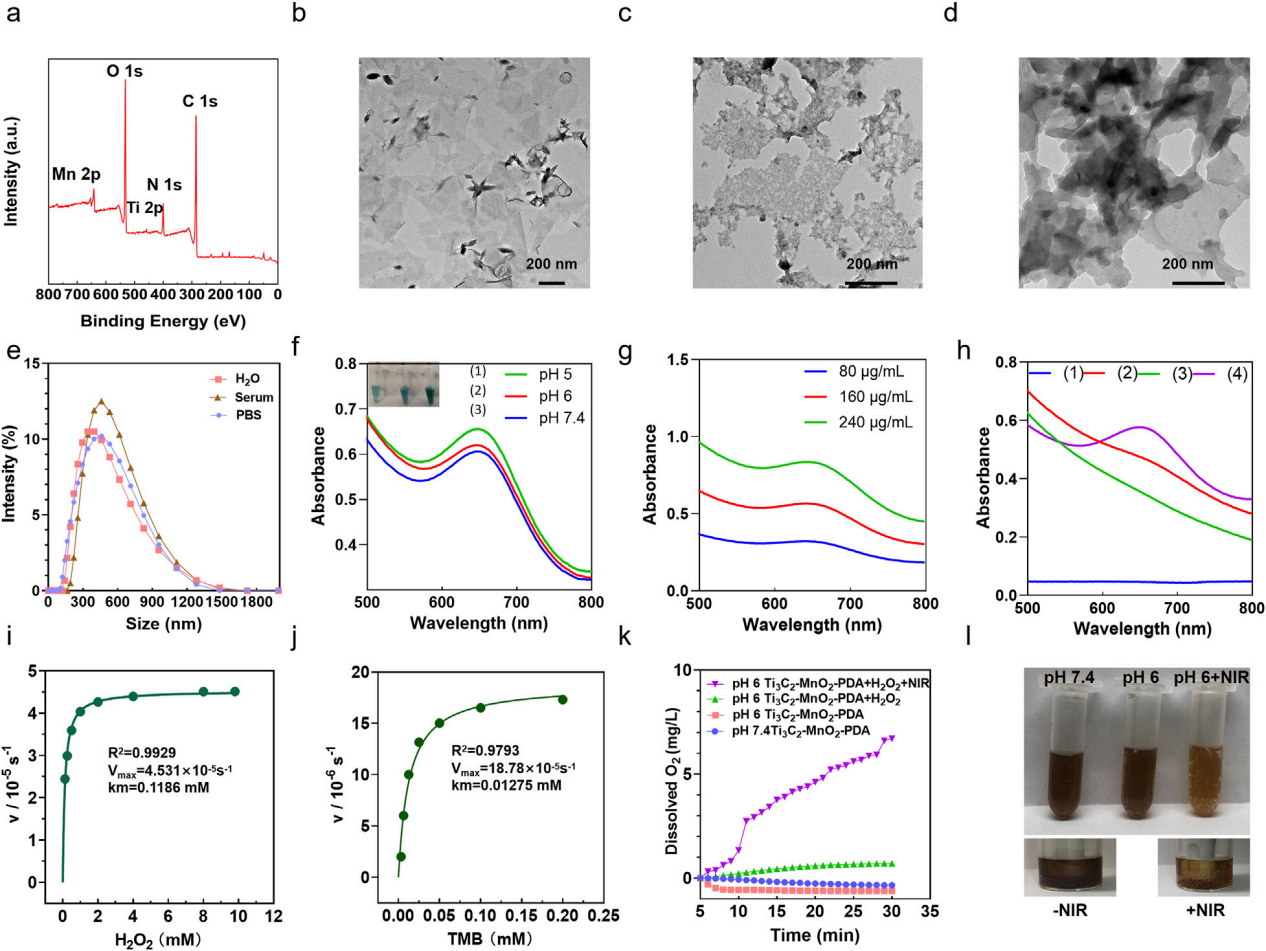
Preparation and characterization of Ti_3_C_2_-MnO_2_-PDA. **(A)** XPS survey spectra of Ti_3_C_2_-MXene-MnO_2_-PDA sample. **(B–D)** Representative TEM images of **(B)** Ti_3_C_2_ (200 nm), **(C)** Ti_3_C_2_-MnO_2_, and **(D)** Ti_3_C_2_-MnO_2_-PDA. **(E)** Particle size of Ti_3_C_2_-MnO_2_-PDA during incubation in H_2_O, PBS, and serum. **(F)** UV-vis absorbance of TMB in the presence of H_2_O_2_ and Ti_3_C_2_-MnO_2_-PDA at different pH levels. Inset shows the corresponding digital photographs of each group. **(G)** UV-vis absorbance of TMB in the presence of H_2_O_2_ and Ti_3_C_2_-MnO_2_-PDA (80 μg/mL, 160 μg/mL, and 240 μg/mL, respectively). **(H)** Absorbance of (1) TMB + H_2_O_2_ (2) TMB + Ti_3_C_2_-MnO_2_-PDA (3) H_2_O_2_ + Ti_3_C_2_-MnO_2_-PDA (80 μg/mL), and (4) TMB + H_2_O_2_ + Ti_3_C_2_-MnO_2_-PDA (80 μg/mL) in the UV-vis region. Ti_3_C_2_-MnO_2_-PDA nanozyme Michaelis−Menten kinetics using **(I)** H_2_O_2_ and **(J)** TMB as the substrates. **(K)** The amount of O_2_ generated by 0.2 μg/mL Ti_3_C_2_-MnO_2_-PDA in the presence of 10 mm H_2_O_2_ at pH 7.4 and pH 6 with or without NIR irradiation. **(L)** Representative images showing O_2_ generation in the Ti_3_C_2_-MnO_2_-PDA, Ti_3_C_2_-MnO_2_-PDA + NIR, and Ti_3_C_2_-MnO_2_-PDA + H_2_O_2_ groups (pH = 7.4, 6.0, respectively).

XPS analyses was employed to determine the elemental compositions and valence states of Ti_3_C_2_-MnO_2_-PDA. The obtained XPS spectra clearly showed the elements of C, Ti, O, Mn and N from PDA modified Ti_3_C_2_-MnO_2_ nanohybrides Figure 1A (Zhou et al., 2019). The high-resolution XPS spectrum of Ti in Supplementary Figure S1 exhibited two peaks located at 458.5 eV and 464.2 eV were designated as Ti-O 2p_3/2_ and Ti-O 2p_1/2_ bonds. The Mn spectrum exhibited two peaks at 652.5 and 641.0 eV, which correspond to the 2p_1/2_ and 2p_3/2_ orbitals of O=Mn=O (Supplementary Figure S2). The N 1s peak at 400 eV which corresponded to the occurrence of N element in survey spectra confirmed the successful modification of PDA film (Yuan et al., 2018; Zhang et al., 2022) (Supplementary Figure S3). Furthermore, the morphology of the Ti_3_C_2_-MnO_2_-PDA nanocomposites was characterized by TEM. As shown in Figure 1B, the Ti_3_C_2_ nanosheets appeared as thin lamella, and the surface of the nanocomposites showed irregular MnO_2_ NPs (Figure 1C), along with a shell coating that corresponded to the PDA layer (Figure 1D). We observed that due to the encapsulation of PDA film, the surface of nanoparticles is smooth, flat, and uniform. At the same time, the nanoparticles are uniformly dispersed in flakes. Although the nanoparticles are slightly adhered after coating modification, they exhibit a certain degree of dispersion overall. When encountering an acidic tumor microenvironment, this PDA membrane responsive rupture can release the internal nanoparticles. In order to better understand the morphology of the nanoparticles, we also demonstrated the nanoparticles at pH 6. Moreover, EDS elemental analysis also demonstrated titanium, manganese and nitrogen in Ti_3_C_2_-MnO_2_-PDA nanoparticles at pH 7.4 (Supplementary Figures S28, 29). Surface modification of Ti_3_C_2_-MXene with PDA significantly improved its stability and dispersion in external and physiological environments (Supplementary Figures S4–S6). Dynamic light scattering measurements of Ti_3_C_2_-MnO_2_-PDA in different media, including water, PBS, and serum, exhibited a consistent diameter of approximately 350 nm (Figure 1E), indicating its good dispersibility. The ζ-potentials of Ti_3_C_2_, Ti_3_C_2_-MnO_2_, and Ti_3_C_2_-MnO_2_-PDA were −20, −36, and −21 mV, respectively (Supplementary Figure S7), which confirmed successful modification with PDA.

### 4.2 Ti_3_C_2_-MnO_2_-PDA exhibits catalytic activity in acidic conditions

Considering the overexpressed H_2_O_2_ and acidic conditions of TME (Uthaman et al., 2018), Ti_3_C_2_-MnO_2_-PDA nanocomposites release MnO_2_ particles following the rupture of the PDA envelope in the acidic TME (Grant et al., 2022), which exhibited both peroxidase (POD)-like and catalase (CAT)-like activity. The POD-like activity of Ti_3_C_2_-MnO_2_-PDA was analyzed using TMB, which is oxidized in the presence of ∙OH and undergoes a colorimetric change. As shown in Figure 1H, TMB solution at 652 nm showed an evident absorption in the presence of Ti_3_C_2_-MnO_2_-PDA and H_2_O_2_. In contrast, the OD_652_ of TMB did not undergo any significant change in the absence of either H_2_O_2_ or Ti_3_C_2_-MnO_2_-PDA, which further confirmed the POD-like activity of the latter. At the same time, we also found that changes in pH can affect the release of ∙OH . As the pH value decreases, the ∙OH released by the nanoenzyme gradually increases, and the TMB color gradually becomes lighter as shown in Figure 1F. We also tested whether the concentration of Ti_3_C_2_-MnO_2_-PDA had an impact on ∙OH production and found that the amount of ∙OH released at pH 6 correlated positively with the nanoenzyme concentration (Figure 1G). Subsequently, the nanoenzymes mediated Fenton-like reaction was evaluated with MB, which could only be degraded by ∙OH (Lin et al., 2018b; Jiang et al., 2022). The absorbance of MB at 670 nm decreases gradually with pH change and nanoenzymes concentration change, which indicates that the released ROS is ∙OH (Supplementary Figure S27). At the same time, we can observe the manganese dioxide nanorods exposed after the gradual rupture of the dopamine membrane when the nano-enzymes are at pH 6, as well as the distribution of Ti, Mn, and N elements in the nano-enzymes Supplementary Figures S28, S29). Furthermore, the kinetic properties of the nanoenzymes were quantitatively analyzed by adjusting the amounts of H_2_O_2_ and TMB, and the Km values and Vmax of the two substrates were obtained by fitting the data through the Michaelis−Menten model and double inverse plotting method (Dang and Zhao, 2020) (Figures 1I, J). The Km value for TMB was 0.01275 mM and the Vmax value was 18.78 × 10^-5^s^−1^. Meanwhile the Km value of H_2_O_2_ was 0.1186 mM and Vmax value was 4.531 × 10^-5^s^−1^. Compared with other POD-like nanoenzymes, the proposed Ti_3_C_2_-MnO_2_-PDA nanoenzymes in this work exhibited lower Km value and higher Vmax (Supplementary Table S1), demonstrating its strong affinity and high POD-like catalytic activity.

To further investigate the CAT-like activity of Ti_3_C_2_-MnO_2_-PDA, we determined the generated O_2_ from mildly acidic (pH 6) and neutral (pH 7.4) H_2_O_2_ solutions catalyzed by Ti_3_C_2_-MnO_2_-PDA and photoirradiation via a dissolved oxygen meter (Figure 1K). As shown in Figure 1L, the non-irradiated solutions did not undergo any noticeable change in amount of produced oxygen regardless of pH, although a few bubbles appeared in the acidic buffer solution. However, a large amount of oxygen was generated and an abundance of gas bubbles was observed in the mixture of Ti_3_C_2_-MnO_2_-PDA and H_2_O_2_ upon NIR irradiation compared with the non-irradiated control, indicating the photo-enhanced activity of this CAT-like nanoenzyme. Consistent with this, NIR irradiation gradually reduced the intensity of the color of acidic buffer, indicating the release of Mn^2+^ from the nanocomposite. The photothermal property of the as-achieved Ti_3_C_2_-MnO_2_-PDA was then evaluated by recording the temperature changes in an aqueous solution. Ti_3_C_2_-MnO_2_-PDA exhibited significant photothermal effects depending on the laser power intensity and its concentration (Supplementary Figures S8, S10), along with good photothermal stability (Supplementary Figure S9) and a high photothermal conversion efficiency of 53.5% (Supplementary Figure S11). These results suggest that Ti_3_C_2_-MnO_2_-PDA can act as a versatile photothermal agent and catalyst for the ROS burst and efficient O_2_ supply to alleviate the hypoxic conditions in the TME (Tang et al., 2021), thus providing a basic environmental condition for the subsequent anti-tumor therapies and the regulation of angiogenesis and immune tolerance (Sun J. et al., 2022; Chen et al., 2023; Gong et al., 2022; Tao et al., 2022; Thienpont et al., 2016).

### 4.3 Ti_3_C_2_-MnO_2_-PDA induces a ROS burst and ICD *in vitro*

**FIGURE 2:**
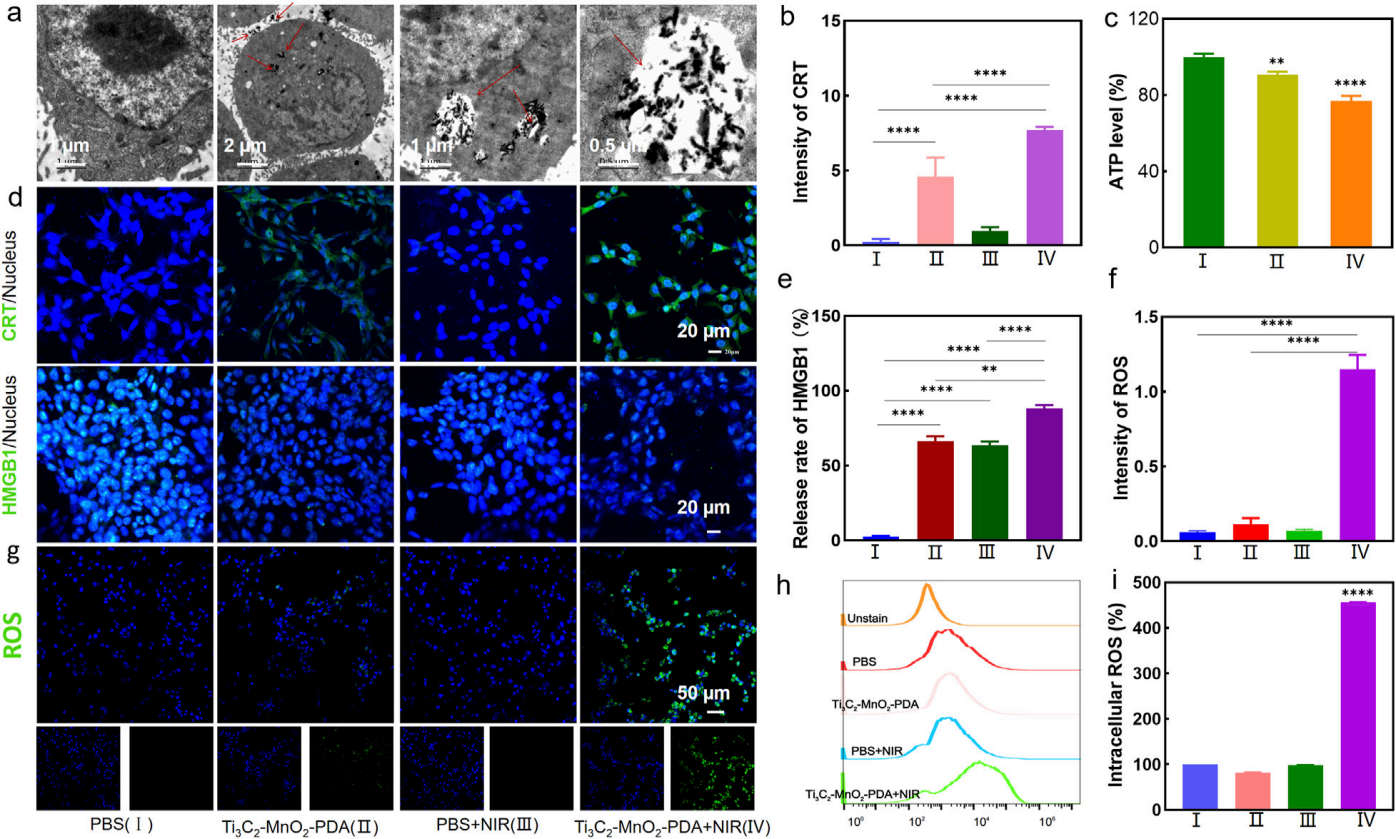
Effector functions of Ti_3_C_2_-MnO_2_-PDA in tumor cells. **(A)** TEM images showing uptake of Ti_3_C_2_-MnO_2_-PDA in tumor cells. **(B, E)** Fluorescence intensities corresponding to the CRT expression and nuclear translocation of HMGB1 in the differentially treated 4T1 cells. **(C)** Intracellular ATP levels in the indicated groups. **(D)** Representative immunofluorescence staining images showing CRT expression and HMGB1 translocation in the differently treated 4T1 cells. Blue and green fluorescence, respectively, indicate the nuclei and CRT/HMGB1 expression (20 μm). **(F)** Fluorescence intensities corresponding to intracellular ROS levels in the indicated groups as measured by enzyme immunoassay. **(G)** Representative fluorescence images showing ROS production in 4T1 cells treated as indicated. **(H, I)** Representative flow cytometry plots showing ROS release in the different treatment groups. Data are expressed as mean ± SD (n = 4). Statistical significance was assessed by a two-tailed Student’s t-test. ***p < 0.001, ****p < 0.0001.

**FIGURE 3:**
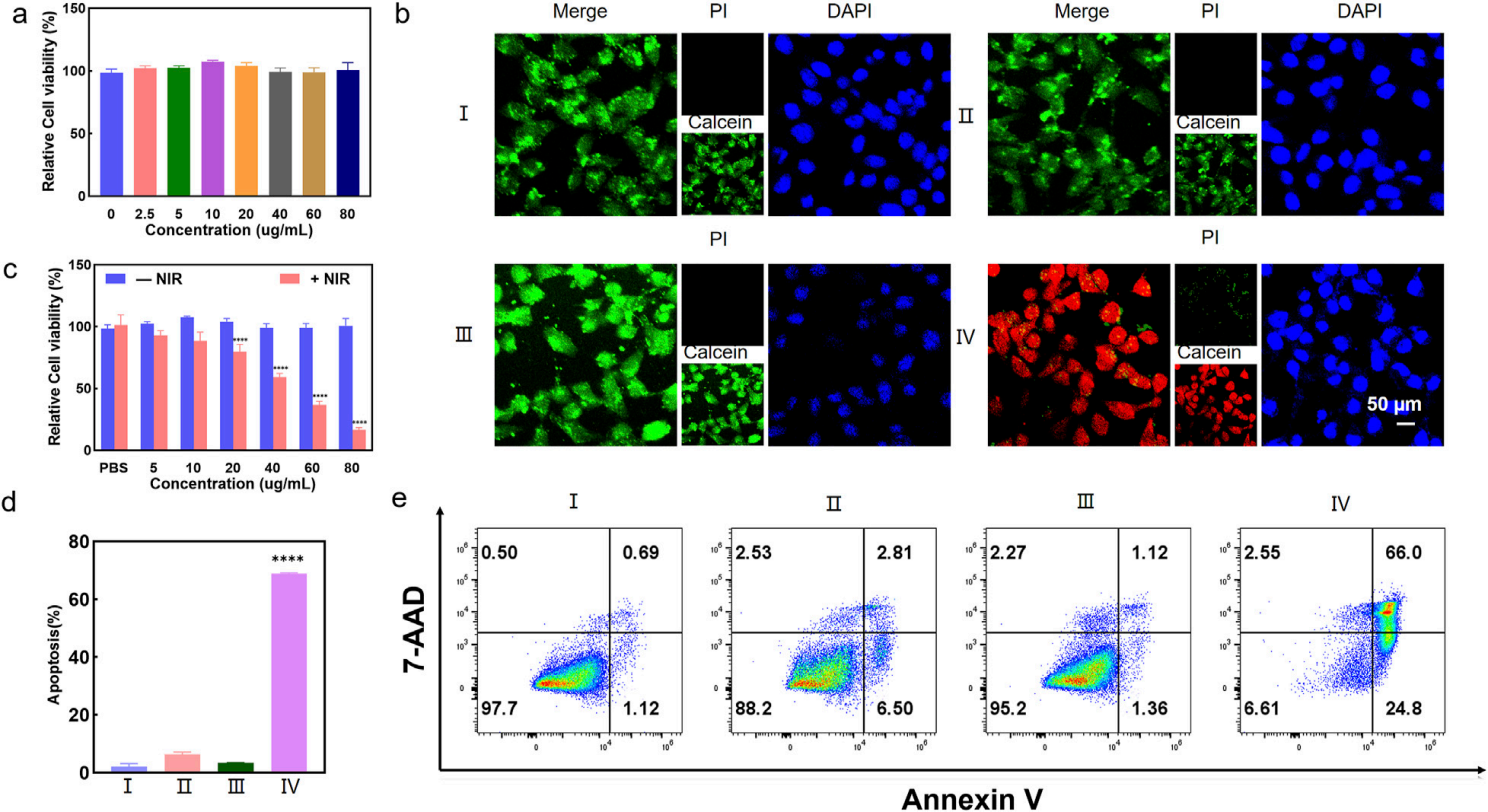
Ti_3_C_2_-MnO_2_-PDA induces a ROS burst and ICD *in vitro*. **(A)** Viability of MCF-10A cells treated with different concentrations of Ti_3_C_2_-MnO_2_-PDA. **(B)** Representative images of calcein-AM/PI staining showing viable and dead cells in the indicated groups. Ⅰ represents PBS group. Ⅱ represents Ti_3_C_2_-MnO_2_-PDA group. Ⅲ represents PBS + NIR group. Ⅳ represents Ti_3_C_2_-MnO_2_-PDA + NIR group.**(C)** Relative viabilities of 4T1 cells treated with different concentrations of Ti_3_C_2_-MnO_2_-PDA with or without NIR irradiation. **(D)** Percentage of apoptotic cells in the indicated groups. **(E)** Representative flow cytometry plots showing annexin V+/7-AAD + apoptotic cells in the indicated groups. Data are expressed as mean ± SD (n = 4). Statistical significance was assessed by a two-tailed Student’s t-test. ****p < 0.0001.

The bio-TEM images in Figure 2A showed that Ti_3_C_2_-MnO_2_-PDA was effectively endocytosed by the 4T1 cells after chemotaxis, guaranteeing the treatment effectiveness. The cytotoxicity of the proposed nanoenzyme was explored prior to *in vitro* experiments. No significant cytotoxicity was observed on normal human breast cells (MCF-10A) treated with Ti_3_C_2_-MnO_2_-PDA, even at high concentration of 80 μg/mL (Figure 3A). Ti_3_C_2_-MnO_2_-PDA decreased the viability of 4T1 cells in a concentration-dependent manner upon laser irradiation (Figure 3B). The viability of the 4T1 cells treated with 40 μg/mL Ti_3_C_2_-MnO_2_-PDA decreased by more than 55% upon laser irradiation (808 nm, 1 W/cm^2^) compared with the non-irradiated control (Figure 3C). Similar results were observed with calcein-AM/PI staining. As illustrated in Figure 3B, the control group exhibited green fluorescence with or without NIR irradiation, demonstrating that NIR irradiation treatment alone had no cytotoxic effect on 4T1 cells. Compared with Ti_3_C_2_-MnO_2_-PDA, widespread 4T1 cell death was observed in the Ti_3_C_2_-MnO_2_-PDA + NIR group characterized by red fluorescence staining with PI. We also examined apoptosis rates in the differentially treated cells using annexin-V/7-AAD staining. The percentage of apoptotic 4T1 cells in the Ti_3_C_2_-MnO_2_-PDA + NIR group reached 90.8% compared with only 1.81% in the PBS control group (Figures 3D, E). These results clearly demonstrate the synergistic therapeutic effect of Ti_3_C_2_-MnO_2_PDA (Sun et al., 2023; Ai et al., 2023; Ming et al., 2022).

To determine whether Ti_3_C_2_-MnO_2_-PDA can induce intracellular ROS production, the 4T1 cells were treated with 60 μg/mL of the nanocomposites for 6 h and irradiated with NIR laser. ROS levels were detected with the DCFH-DA probe. As shown in Figure 2G, the irradiated cells emitted stronger fluorescence signals compared with the non-irradiated cells, indicating higher ROS production. In contrast, no ROS production was observed in the PBS control, suggesting laser irradiation itself did not induce ROS production. Consistent with the above findings, flow cytometric analysis showed 5-fold higher ROS levels in the Ti_3_C_2_-MnO_2_-PDA + NIR group compared with the control group (Figures 2H, I). We also detected ROS production via an enzyme immunoassay, and the results indicated a 1.2-fold increase in the fluorescence intensity of ROS in the Ti_3_C_2_-MnO_2_-PDA + NIR group, compared with the control group (Figure 2F). Taken together, photothermal and the catalytic activity of Ti_3_C_2_-MnO_2_-PDA synergistically triggered a ROS burst in the tumor cells, and the ensuing oxidative stress induced apoptosis via mitochondrial dysfunction (Tu et al., 2021).

The ROS burst was expected to induce ICD and subsequently an anti-tumor immune response. To this end, we analyzed the expression of the ICD markers, CRT and HMGB1, and then measured the intracellular ATP levels using a chemiluminescent probe. The 4T1 cells in the Ti_3_C_2_-MnO_2_-PDA + NIR group showed a significant increase in the expression of CRT in their membranes (Figures 2B, D), along with the translocation of HMGB1 from the nucleus to the cytoplasm (Figure 2E), which were indicative of ICD. Furthermore, ATP levels decreased significantly following treatment with Ti_3_C_2_-MnO_2_-PDA and NIR irradiation (Figure 2C), which can be explained by the high levels of oxygen produced by the catalytic action of Ti_3_C_2_-MnO_2_-PDA, leading to increased glucose consumption and starvation effects that eventually decreased ATP levels within the tumor. Ti_3_C_2_-MnO_2_-PDA induces ICD, which promotes the maturation of dendritic cells (DCs) and triggers a tumor-specific T cell immunity response that can alleviate an immunosuppressive microenvironment.

### 4.4 Ti_3_C_2_-MnO_2_-PDA inhibits tumor growth and metastasis *in vivo*

**FIGURE 4:**
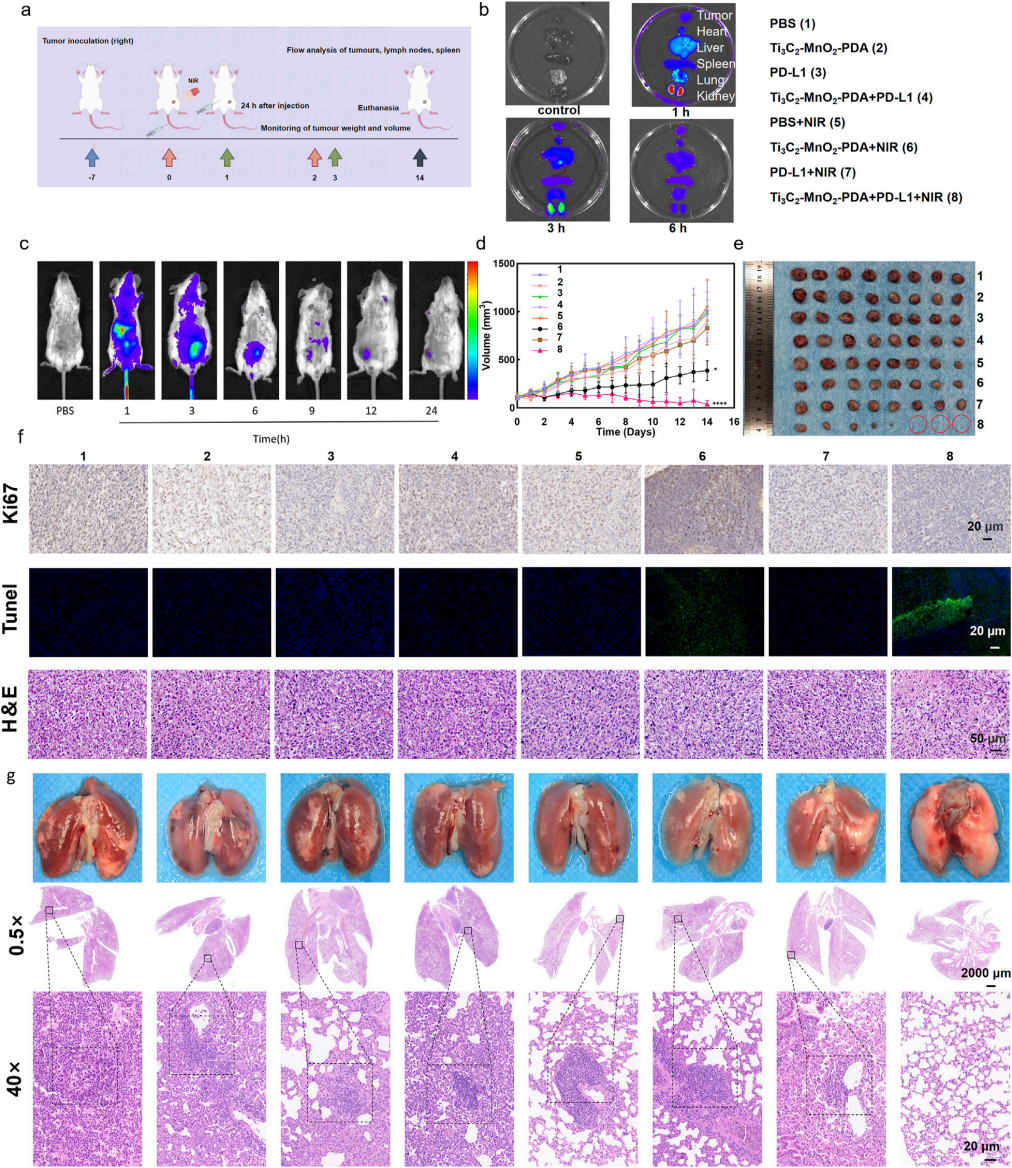
Ti_3_C_2_-MnO_2_-PDA inhibits tumor growth and metastasis *in vivo*. **(A)** Schematic illustration of the establishment of the 4T1 tumor model and treatment protocol. On day 7, BALB/c mice are intravenously injected with Ti_3_C_2_-MnO_2_-PDA and irradiated with NIR laser (1 W/cm^2^, 5 min) 1 h later. Anti-PD-L1 antibody is injected intraperitoneally 24 h later. (By Figdraw). **(B, C)** Representative *ex vivo* and *in vivo* fluorescence images of the tumors and major organs at different time points following injection of Ti_3_C_2_-MnO_2_-PDA-Cy5(n = 3). **(D)** Tumor volume treated with different strategies. **(E)** Representative images of tumors isolated on day 14 from the indicated groups. **(F)** Representative images of H&E (50 μm), Ki67, and TUNEL (20 μm) staining of tumor tissues from the indicated groups. Data was expressed as mean ± SEM n = 8). **(G)** Representative images of lungs with metastatic nodules (top) and H&E-stained lung sections (2000 μm) from the indicated groups. The black dotted lines demarcate the metastatic tumor areas (20 μm). Data was expressed as mean ± SEM (n = 8). Statistical significance was assessed by a two-tailed Student’s t-test. **p < 0.01, ***p < 0.001, ****p < 0.0001.

The anti-tumor effects of Ti_3_C_2_-MnO_2_-PDA observed *in vitro* were further validated in a murine model of breast cancer. To analyze the *in vivo* pharmacokinetics of the nanocomposites, each BALB/c mouse was intravenously injected with 100 μL of Cy5-labeled Ti_3_C_2_-MnO_2_-PDA (10 mg/kg), and the fluorescence signals in the tumor site and other major organs (heart, liver, spleen, lung, and kidney) were detected at different time points after injection guided by a small-animal imager. The fluorescence intensity at the tumor site peaked within 1 h after injection and remained higher than that of other organs at 3 h (Figures 4B, C; Supplementary Figure S15), indicating effective accumulation of Ti_3_C_2_-MnO_2_-PDA in the tumor tissues. It was also observed that the dopamine-encapsulated nano-enzymes were mainly metabolised and excreted through the liver and kidneys. After 24 h, only a small amount of fluorescent signal was seen at the tumour site, suggesting that the nano-enzymes have a good retention effect and tumour tropism at the tumour site, whereas they would be cleared very quickly in the normal organs, reducing the impact on the body. To evaluate the potential therapeutic effects of Ti_3_C_2_-MnO_2_-PDA, the tumor-bearing mice were subjected to different treatment protocols (Figure 4A) once the tumor diameter reached approximately 100 mm^3^, and the outcomes were evaluated on day 14 post-treatment. The tumor volume and weight were significantly reduced in mice treated with Ti_3_C_2_-MnO_2_-PDA + NIR irradiation compared with the control groups. As anticipated, introduction of the anti-PD-L1 antibody significantly inhibited tumor growth compared to that seen with single Ti_3_C_2_-MnO_2_-PDA upon NIR irradiation (Figures 4D, E, Supplementary Figures S16, S17a), most likely because of stronger immune activation following immune checkpoint blockade. Furthermore, H&E staining of the tumor tissues revealed that combination treatment with Ti_3_C_2_-MnO_2_-PDA and anti-PD-L1 largely restored the nuclear morphology in the malignant cells. Consistent with the evident decrease in tumor growth, the proportion of Ki-67^+^ proliferative cells was significantly lower, whereas that of TUNEL^+^ apoptotic cells was higher in the Ti_3_C_2_-MnO_2_-PDA + anti-PD-L1 group compared with that in other treatment groups (Figure 4F). The potential toxicity of Ti_3_C_2_-MnO_2_-PDA was evaluated on the basis of histopathological findings and serum biochemical indices, showing no significant differences among the treatment groups (Supplementary Figures S13, S18, S20). In addition, no distinct changes were observed in the body weight of mice in any of the groups (Supplementary Figure S17b). These results confirmed the excellent biosafety of Ti_3_C_2_-MnO_2_-PDA.

To evaluate the impact of the combination treatment on metastasis, we established a lung metastasis model by inoculating female BALB/c mice with 4T1 cells *in-situ*. The mice were euthanized 29 d later, and the metastatic nodules were counted and processed for histopathological examination using H&E. The number of lung metastatic nodules was significantly reduced in the Ti_3_C_2_-MnO_2_-PDA + NIR group compared with the other groups that exhibited massive metastatic regions (Figure 4G). Furthermore, the lung tissues in the Ti_3_C_2_-MnO_2_-PDA + NIR + anti-PD-L1 group demonstrated no metastatic nodules, indicating the remarkable suppression of lung metastasis with our strategy. Consistent with this, significant differences in the lung weight were observed among the treatment groups (Supplementary Figure S26). A remarkable pulmonary lymph node metastasis rate (Supplementary Figure S25) toward 4T1 tumor metastasis was also observed in the Ti_3_C_2_-MnO_2_-PDA + NIR treatment, indicating the outstanding anti-tumor immune effect of the nanoenzyme itself. Overall, anti-PD-L1 assisted Ti_3_C_2_-MnO_2_-PDA in promoting a significant inhibitory effect on primary tumor growth, as well as lung metastasis.

### 4.5 The combination of Ti_3_C_2_-MnO_2_-PDA and anti-PD-L1 blockade enhances the anti-tumor immune response

**FIGURE 5:**
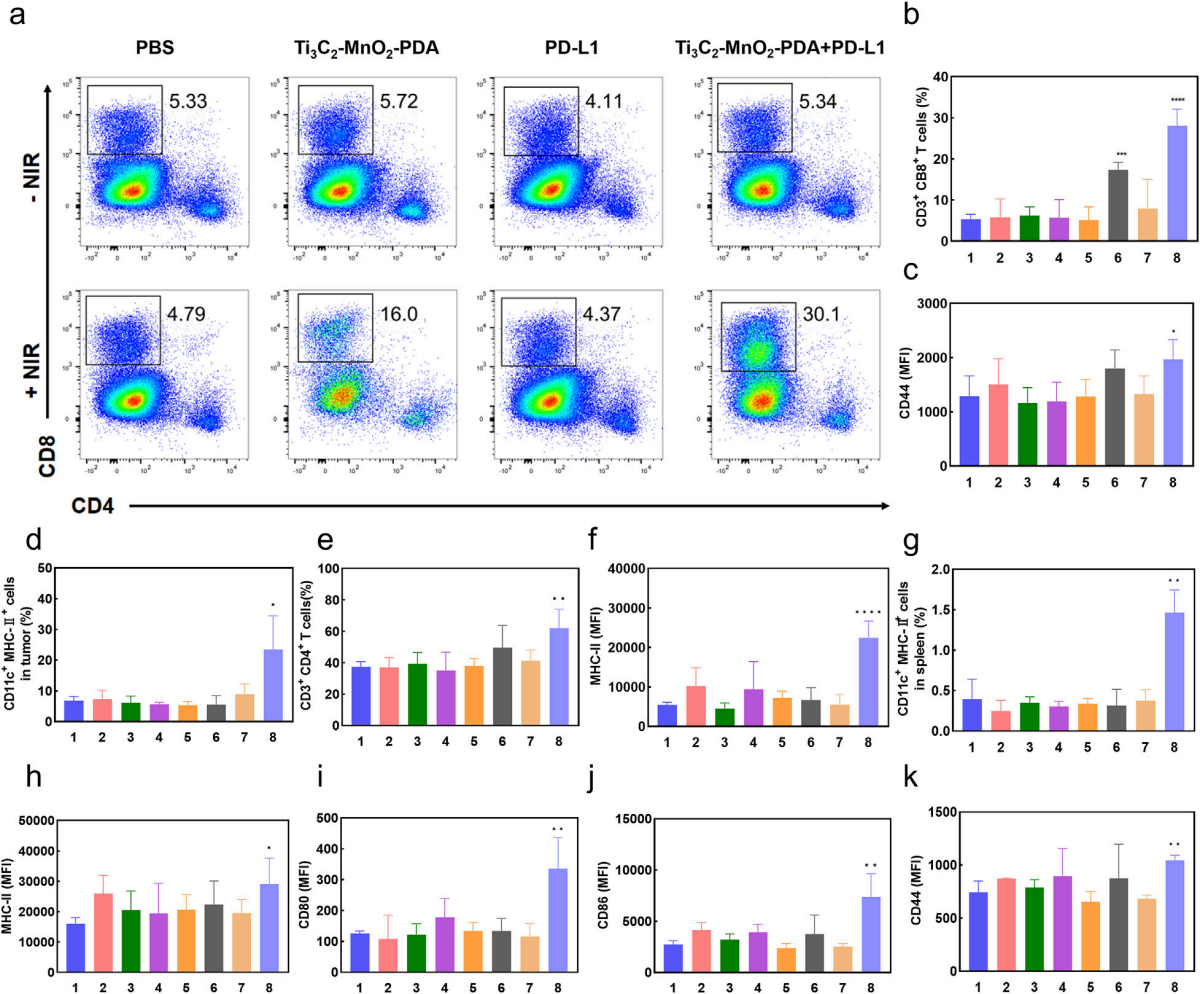
Ti_3_C_2_-MnO_2_-PDA reverses immunosuppression in the TME. **(A)** Flow cytometry plots showing CD4^+^ T cells and CD8^+^ T cells in the tumors of mice from the indicated groups. **(B–K)** Bar graphs showing **(B)** proportion of the CD3^+^/CD8^+^ T cells and **(C)** CD44^+^ CD8^+^ T cells in tumor tissues, **(D)** proportion of activated DCs in tumor tissues, **(E)** proportion of CD4^+^/CD3^+^ T cells in the ipsilateral tumor draining lymph nodes, **(F)** MHC-II expression in the lymph node DCs, **(G)** proportion of DCs in the spleen, **(H–J)** expression of CD80, CD86, and MHC II in splenic DCs, and **(K)** expression of CD44 in splenic CD8^+^ T cells in the indicated groups. *p < 0.05, **p < 0.01, ***p < 0.001, ****p < 0.0001 relative to the PBS group.

Extensive evidence has highlighted the crucial roles of the TME in cancer immunotherapy, and the achieved anti-tumor efficiency of the proposed therapy strategy inspired us to assess the immunomodulatory effects of Ti_3_C_2_-MnO_2_-PDA in the TME. Consequently, we analyzed the changes in immune cell populations in the tumor tissues, spleen, and ipsilateral tumor draining lymph nodes using flow cytometry. The proportion of CD8^+^ T cells in the tumor-infiltrating leukocytes was greatly increased in the Ti_3_C_2_-MnO_2_-PDA + NIR and Ti_3_C_2_-MnO_2_-PDA + anti-PD-L1 + NIR groups (Sun et al., 2023; Ai et al., 2023; Ming et al., 2022; Xia et al., 2024). These references mainly illustrate the mechanisms of activation of CD8^+^ Tcell immunity by photothermal therapy as well as by combined application in combination with anti-PD-L1 antibodies, as well as the underlying signalling pathways, which are the therapeutic options applied in this manuscript. Furthermore, the combination therapies also increased the expression of CD44 in the CD8^+^ T cells, which is indicative of T cell activation and subsequent anti-tumor immune response (Figures 5A–C). The gating strategies of the other immune cell subpopulations are shown in Supplementary Figures S21–S24. DCs are the predominant antigen-presenting cells that play a key role in T cell activation. We found that the number of intra-tumoral DCs was significantly higher in the Ti_3_C_2_-MnO_2_-PDA + anti-PD-L1 + NIR group (Figure 5D), indicating that the combination of Ti_3_C_2_-MnO_2_-PDA and anti-PD-L1 effectively promoted DC recruitment and T cell activation. Furthermore, PD-L1 blockade effectively reduced immune escape, which further enhanced the local infiltration of the CD8^+^ cytotoxic T lymphocytes. Indeed, the tumor tissues in this group showed high *in-situ* expression of CRT and HMGB1, which was consistent with the *in vitro* findings, confirming that Ti_3_C_2_-MnO_2_-PDA + NIR treatment induced localized ICD in the tumor (Supplementary Figure S19).

It has been shown that manganese (Mn^2+^) is a STING activator, which can directly activate cGAS and enhance the binding affinity between cGAMP and STING, thus stimulating the activation of CD8 T and NK cells. Delivery of Mn^2+^ to DC cells in the spleen by intravenous injection is expected to significantly enhance DC maturation and antigen presentation in the spleen, thus rapidly activating the anti-cancer immunity of surrounding T cells (Luo Z. et al., 2024). Thus, the presence of Mn^2+^ in the TME can significantly enhance the efficacy of anti-tumor immunotherapy. We detected an increased proportion of CD4^+^ T cells (Figure 5E, Supplementary Figure S24), as well as high major histocompatibility complex II (MHC II) expression on the DCs (Figure 5F) in the ipsilateral tumor draining lymph nodes, which were indicative of successful antigen presentation to specific T cells and their activation. The splenocytes in the Ti_3_C_2_-MnO_2_-PDA + anti-PD-L1 + NIR group also had a higher proportion of CD44^+^ CD8^+^ T cells (Figure 5K) and DCs (Figure 5G). Furthermore, increased expression of CD80, CD86, and MHC II in the splenic DCs was indicative of an activated state (Figures 5H–J). Taken together, the combination of Ti_3_C_2_-MnO_2_-PDA and anti-PD-L1 blockade not only increased the infiltration of immune cells in the tumor tissues and reversed the immunosuppressive microenvironment but also activated the peripheral immune system, resulting in synergistic anti-tumor action.

## 5 Conclusion

In this study, we successfully fabricated a TME-responsive artificial nanoenzyme with POD and CAT activity. In virtue of high-level H_2_O_2_ and the acidic TME, the photo-enhanced CAT/POD activity and photothermal effect of Ti_3_C_2_-MnO_2_-PDA enabled chemodynamic and thermal ablation of the tumor cells with a simultaneous supplement of oxygen. Further combination of Ti_3_C_2_-MnO_2_-PDA nanoenzyme with PD-L1 checkpoint blockade resulted in the synergist inhibition of primary and metastatic tumor growth. Subsequent assessment of the immunogenic TME demonstrated that this combined PTT-PDT-immunotherapy strategy would inhibit tumor growth and exhibit an abscopal effect primarily by reversing the immunosuppressive microenvironment and activating the peripheral immune system. The current potential of nanoenzymes in medicine cannot be ignored, but many challenges need to be overcome to realise their widespread clinical use. For example, the effect of changes in preparation conditions of nanoenzymes on the morphology, size and catalytic activity of the enzyme. There is a lack of uniformity in the process of nanoenzymes preparation. Therefore, the establishment of a standardised preparation process and a standardised evaluation system is the key to promote the clinical application of nanoenzymes. Our work proposes a multifunctional theranostic platform that is responsive to and capable of modulating the TME, holding great promise for anti-tumor immunotherapy. This provides new ideas for the development of nano-enzymatic anti-tumour immunotherapy.

## Data Availability

The original contributions presented in the study are included in the article/Supplementary Material, further inquiries can be directed to the corresponding author.
