# Supplementary material for: Tumor microenvironment–responsive and modulatory manganese-based nanoenzyme for enhanced tumor immunotherapy
Source: Front Pharmacol. 2025 Jan 3;15:1518983. doi: 10.3389/fphar.2024.1518983 (PMC11739168; doi:10.3389/fphar.2024.1518983)
Supplement: Supplementary file 1 [file DataSheet1.docx]

**Tumor microenvironment–responsive and modulatory manganese-based nanoenzyme for enhanced tumor immunotherapy**

Qi Yang^a, #^, Qiong Wu^b, #^, HaiYan Liu^c^, JianDong Wu^b^, FengMa^d^, XiaoFeng Tian^e, *^

^a^Department of 1 Gynecology and Obstetrics, China-Japan Union Hospital of Jilin University, 126 Sendai Street, Changchun 130033, Jilin, China

^b^Key Laboratory of Pathobiology, Ministry of Education, Nanomedicine and Translational Research Center, China-Japan Union Hospital of Jilin University, 126 Sendai Street, Changchun 130033, Jilin, China

^c^Key Laboratory of Pathobiology Ministry of Education, Department of Anatomy, College of Basic Medical Sciences, Jilin University, Changchun 130061, China

^d^Department of Pathology, China-Japan Union Hospital of Jilin University, 126 Sendai Street, Changchun 130033, Jilin, China

^e^Department of General Surgery, China-Japan Union Hospital of Jilin University, 126 Sendai Street, Changchun 130033, Jilin, China

Corresponding author email: txf@jlu.edu.cn (XiaoFeng Tian)

^#^The authors contributed equally to this work.


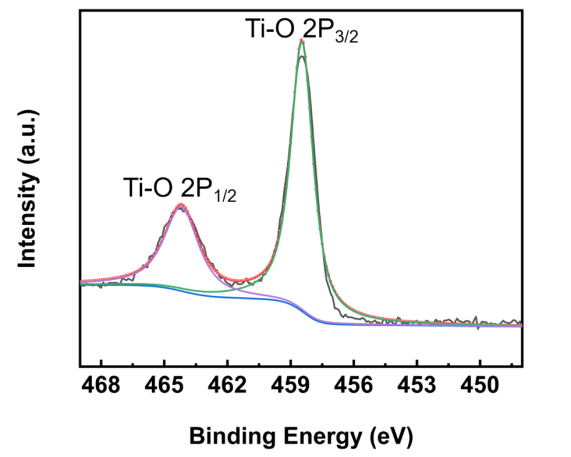


Figure S1.High-resolution XPS spectra of Ti in the Ti_3_C_2_-MnO_2_-PDA nanocomposites.


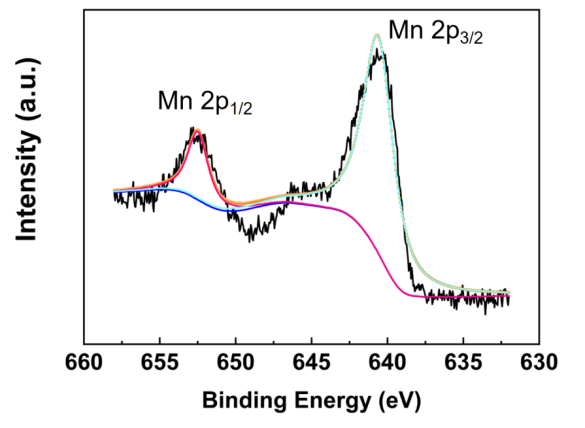


Figure S2.High-resolution XPS spectra of Mn in the Ti_3_C_2_-MnO_2_-PDA nanocomposites.


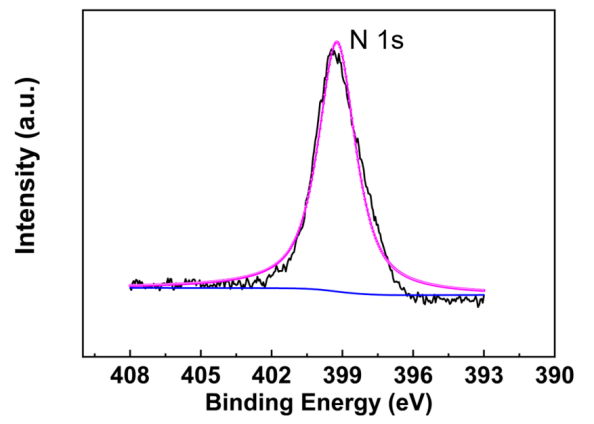


Figure S3.High-resolution XPS spectra of N in the Ti_3_C_2_-MnO_2_-PDA nanocomposites.


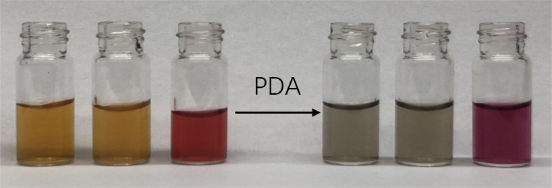


Figure S4. Photos of Ti_3_C_2_-MnO_2_ and Ti_3_C_2_-MnO_2_-PDA in water, PBS, and RPMI medium solution.

Figure S5. UV-vis absorption of Ti_3_C_2,_ Ti_3_C_2_-MnO_2_ nanosheets, and Ti_3_C_2_-MnO_2_-PDA nanocomposites.

Figure S6. UV-vis absorption spectra of Ti_3_C_2_-MnO_2_-PDA nanocomposites before and after storage for 1 month.

Figure S7. Zeta potentials of Ti_3_C_2_-MXene nanosheets, Ti_3_C_2_-MnO_2_ and Ti_3_C_2_-MnO_2_-PDA nanocomposites.

Figure S8.Temperature changes in Ti_3_C_2_-MnO_2_-PDA solutions of different concentrations in response to NIR laser irradiation and relationship between the temperature of 40 μg/mL Ti_3_C_2_-MnO_2_-PDA solution and laser power density.

Figure S9. Thermal stability measurement of Ti_3_C_2_-MnO_2_-PDA (40 μg/mL) undergoing continuous four cycles of laser on/off (1.0 W/cm^2^ ).


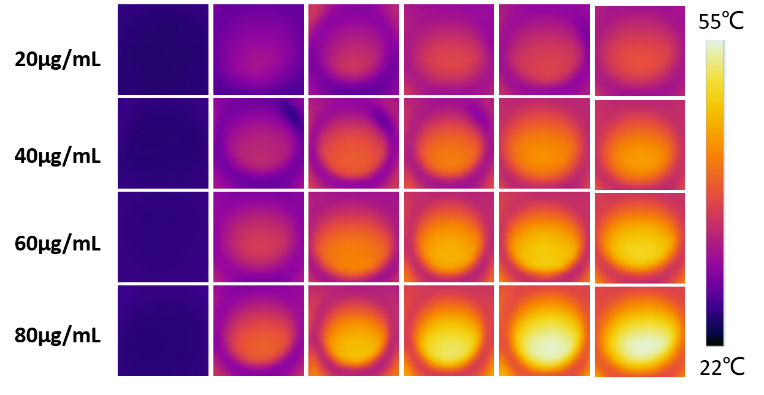


Figure S10. Thermal images of Ti_3_C_2_-MnO_2_-PDA at various concentrations upon laser irradiation


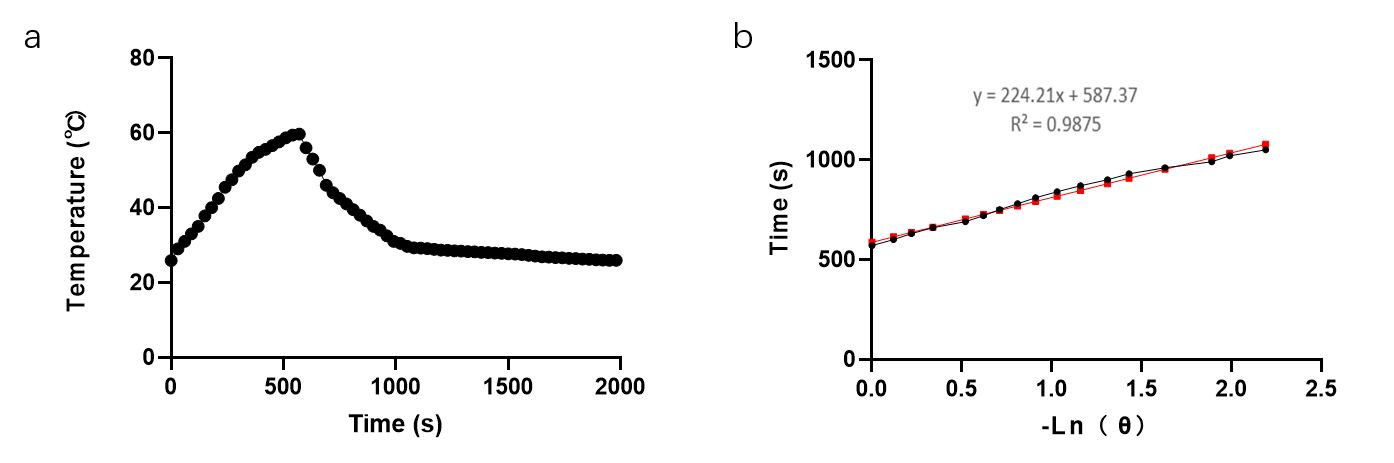


Figure S11. Photothermal conversion ability of Ti_3_C_2_-MnO_2_-PDA. (a) The photothermal effect of the Ti_3_C_2_-MnO_2_-PDA (0.16 mg/mL) under 808 nm laser irradiation and shutdown (1.0 W/cm^2^). (b)The cooling period plot against the negative natural logarithm of the temperature according to the cooling stage of Figure.

Figure S12. Relative viabilities of MDA-MB-231 cells after co-incubation with different concentrations of Ti_3_C_2_-MnO_2_-PDA under 808 nm laser treatment (1.5 W/cm^2^). Data were expressed as mean ± SD (n=3). ****p < 0.0001.


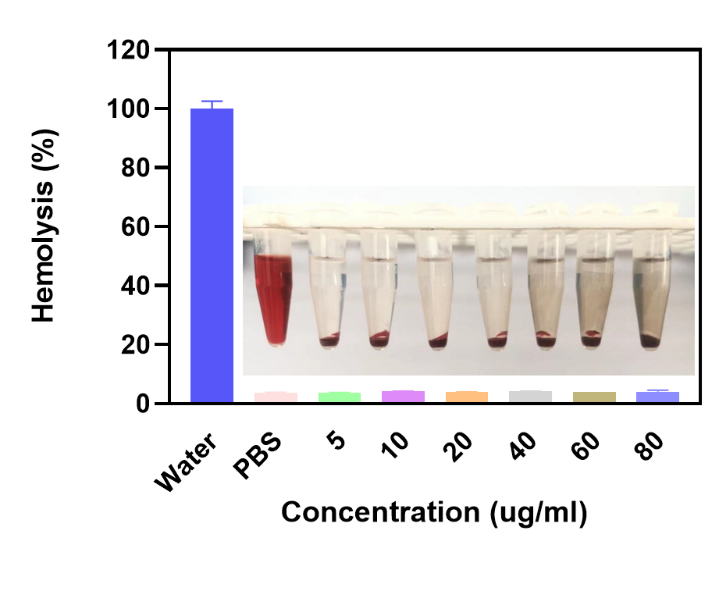


Figure S13. Hemolysis images of RBCs treated with H_2_O, PBS and Ti_3_C_2_-MnO_2_-PDA at 5 μg/mL,10 μg/mL, 20 μg/mL, 40 μg/mL, 60 μg/mL. and 80 μg/mL.


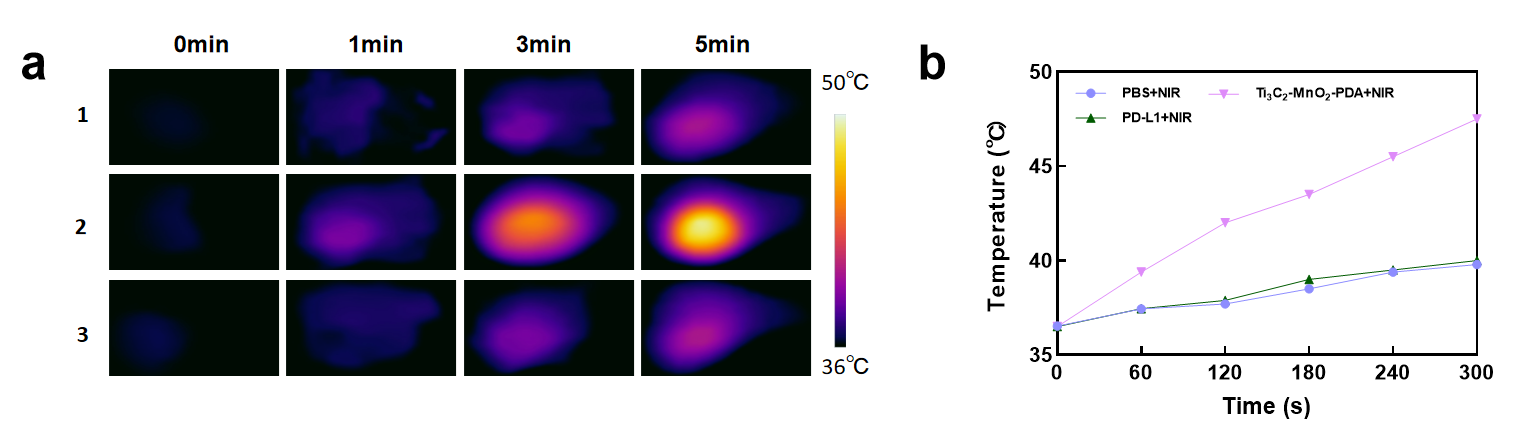


Figure S14. (a) Infrared thermal images of tumors in mice with different treatments. (1) PBS + NIR;(2) Ti_3_C_2_-MnO_2_-PDA+NIR; (3) PD-L1 + NIR. (b) The temperature changes at the tumor sites of 4T1-tumor-bearing mice during local NIR irradiation corresponding to the photothermal imaging of mice.

Figure S15. Fluorescence intensity statistics of tumour sites at different time points in mice injected intravenously with Ti_3_C_2_-MnO_2_-PDA-Cy5 in vivo.


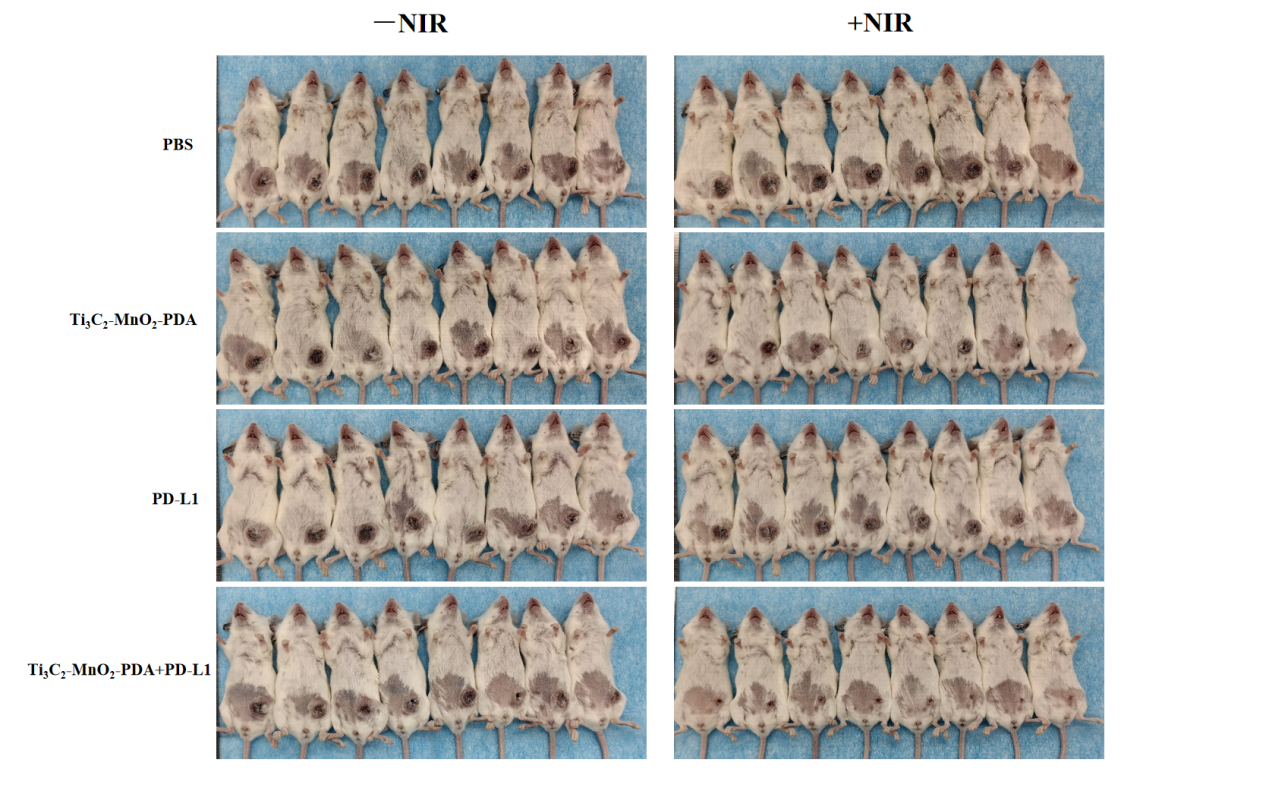


Figure S16. Individual images of 4T1 tumor-bearing mice from different treatment groups at day 14. Group 1, PBS; Group 2, Ti_3_C_2_-MnO_2_-PDA; Group 3, PD-L1; Group 4, Ti_3_C_2_-MnO_2_-PDA + PD-L1; Group 5, PBS+NIR; Group 6, Ti_3_C_2_-MnO_2_-PDA +NIR; Group 7, PD-L1+NIR; Group 8, Ti_3_C_2_-MnO_2_-PDA + PD-L1+NIR.


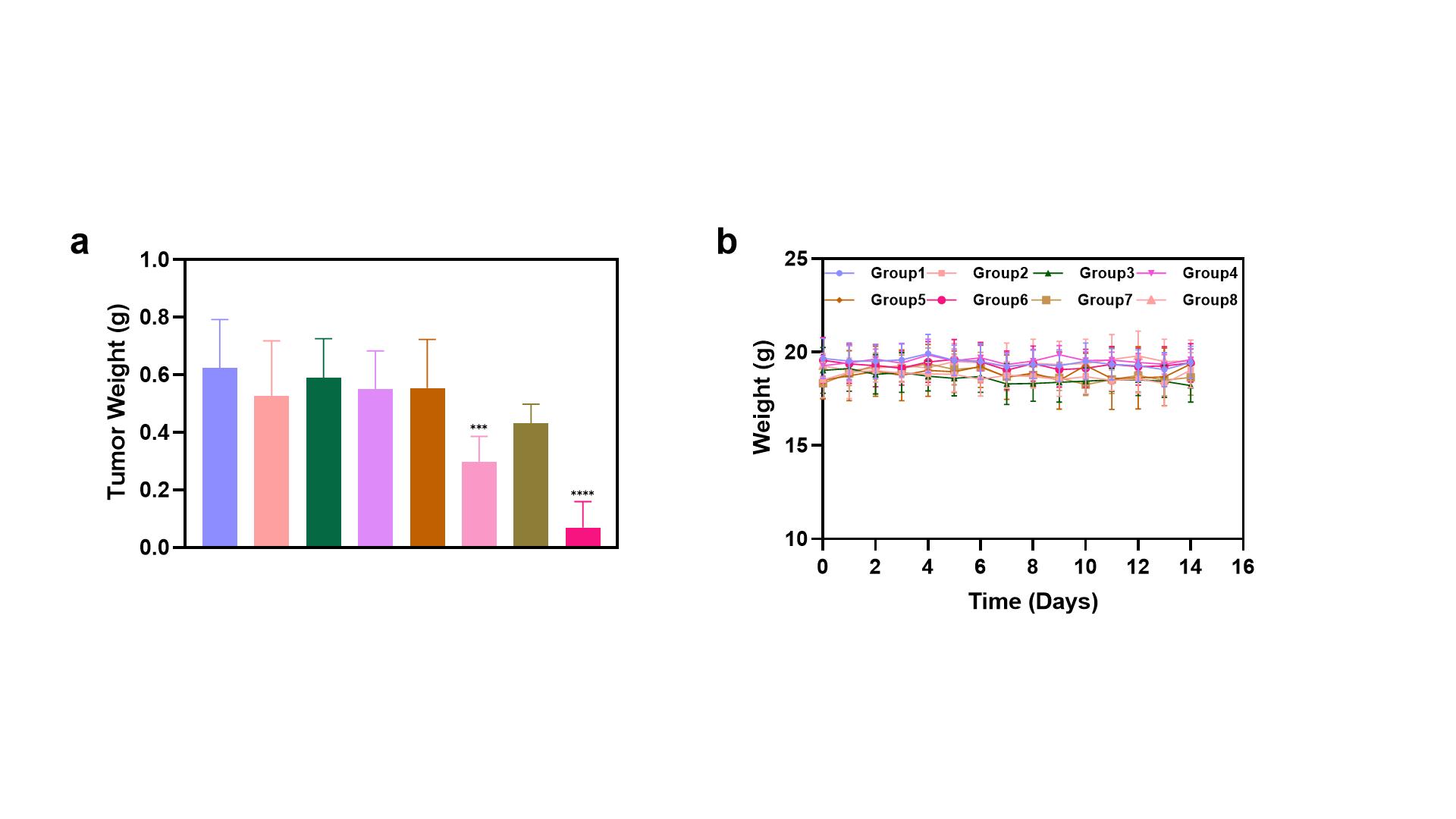


Figure S17. (a) Quantification of tumor weight on day 14 and (b) Body weights.


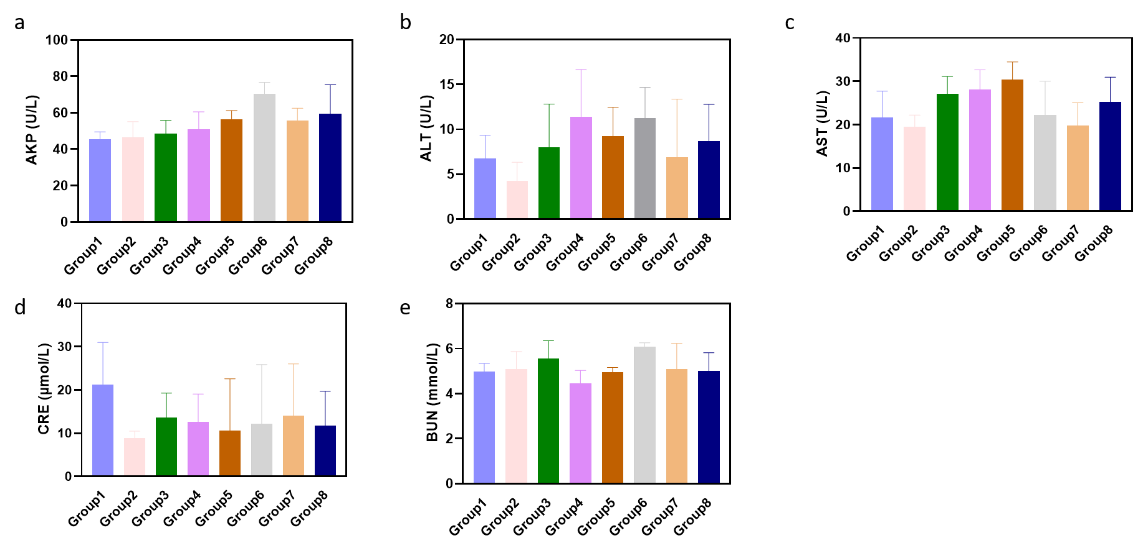


Figure S18. The levels of the serum liver function markers AKP (a), ALT (b), and AST (c), and the levels of the serum kidney function markers CRE (d) and BUN (e). Group1, PBS; Group 2, Ti_3_C_2_-MnO_2_-PDA; Group 3, PD-L1; Group 4, Ti_3_C_2_-MnO_2_-PDA+PD-L1; Group 5, PBS+NIR; Group 6, Ti_3_C_2_-MnO_2_-PDA+NIR; Group 7, PD-L1+NIR; Group8, Ti_3_C_2_-MnO_2_-PDA+PD-L1+NIR


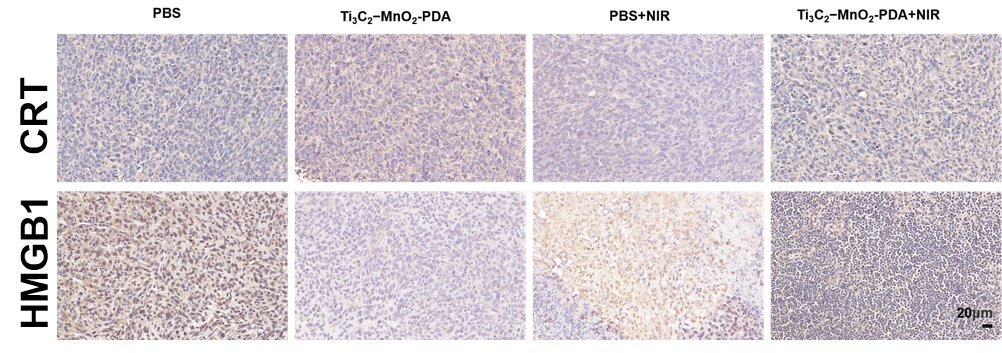


Figure S19. The CRT and HMGB1 immunohistochemical staining images of tumor biopsies from different treatment groups (20 μm).


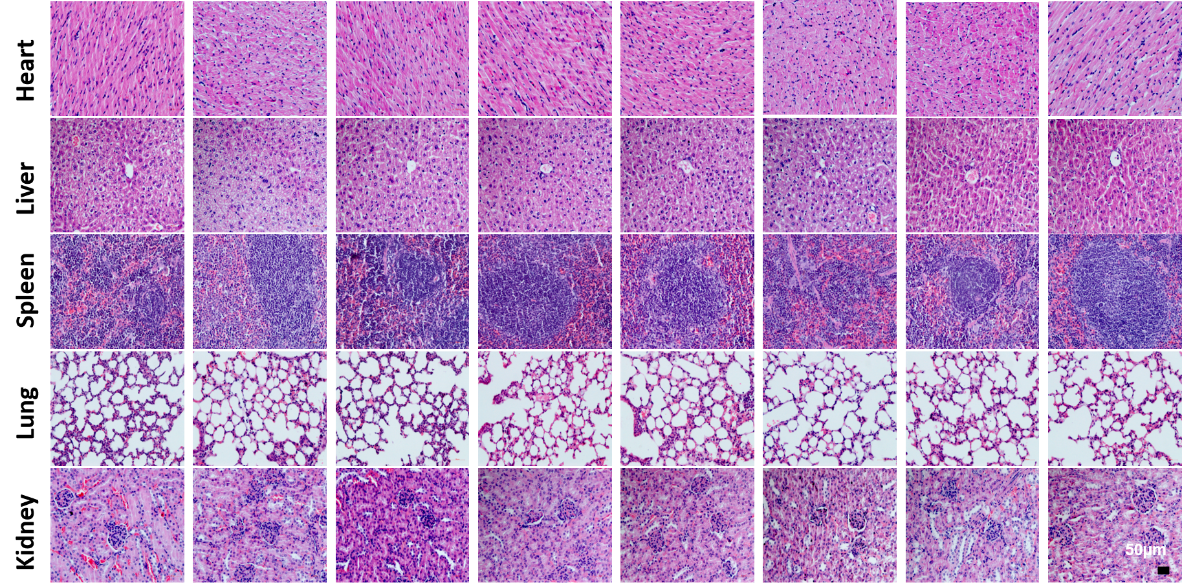


Figure S20.H&E images of different tissues in BALB/C mice in different groups (50 μm).


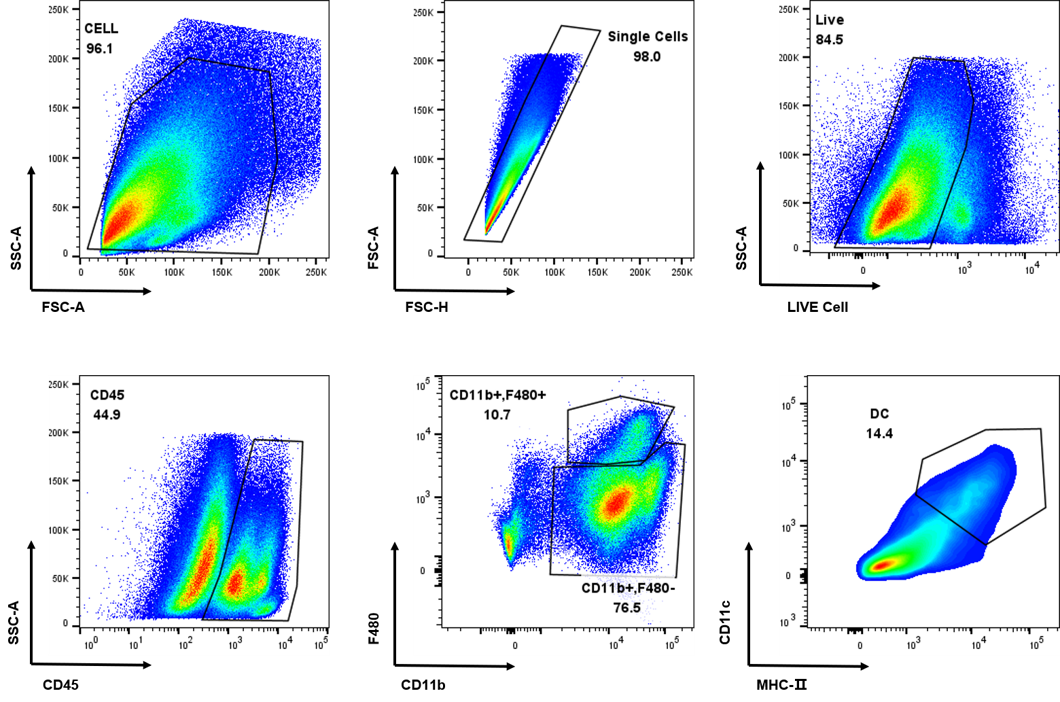


Figure S21. Gating strategy for activation DCs analysis in the tumor.


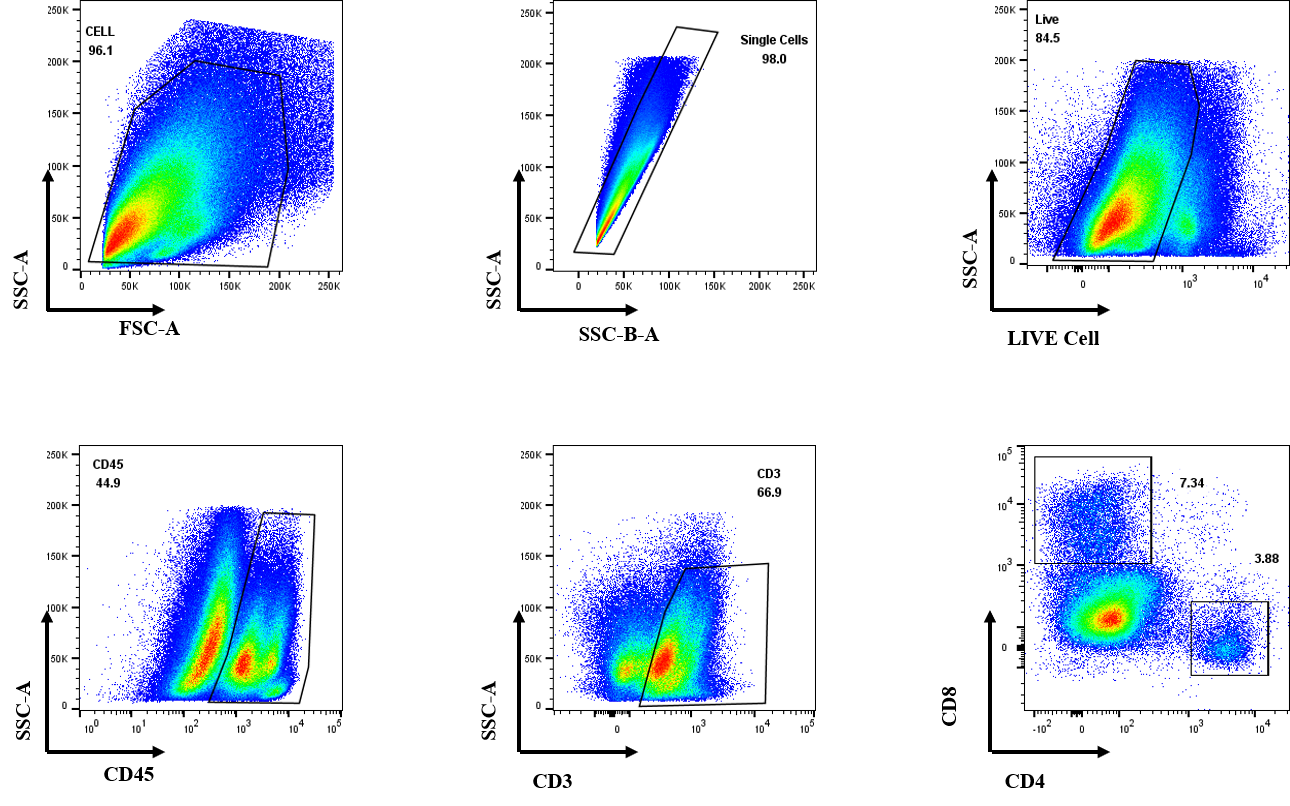


Figure S22. Gating strategy for CD4^+^ and CD8^+^ T cells analysis.


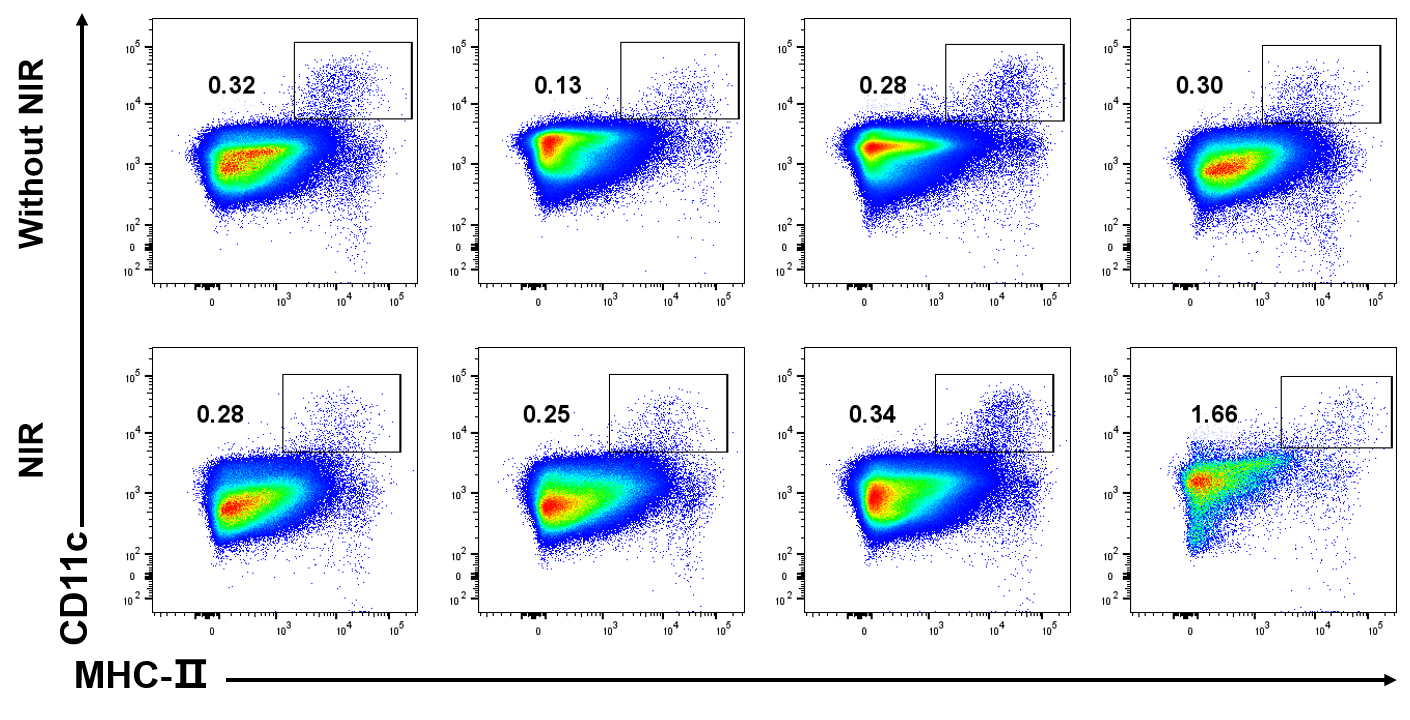


Figure S23. Flow cytometric analyses of the populations of DC in the spleen.


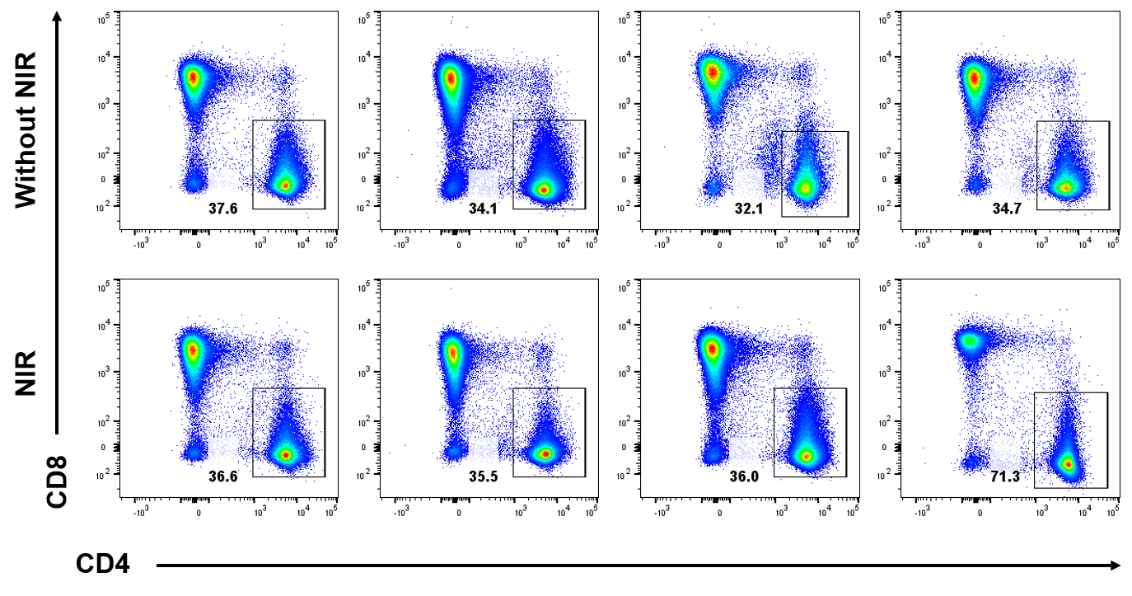


Figure S24. Flow cytometric analyses of the populations of CD4^+^ T cells in lymph.


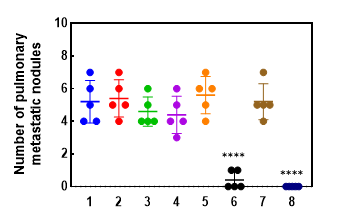


Figure S25. Number of metastatic lung nodules in the indicated groups (n = 5). *p < 0.05, **p < 0.01, ***p < 0.001, ****p < 0.0001


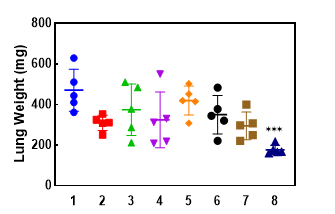


Figure S26. Lung tissue weight in the indicated groups (n = 5). *p < 0.05, **p < 0.01, ***p < 0.001, ****p < 0.0001


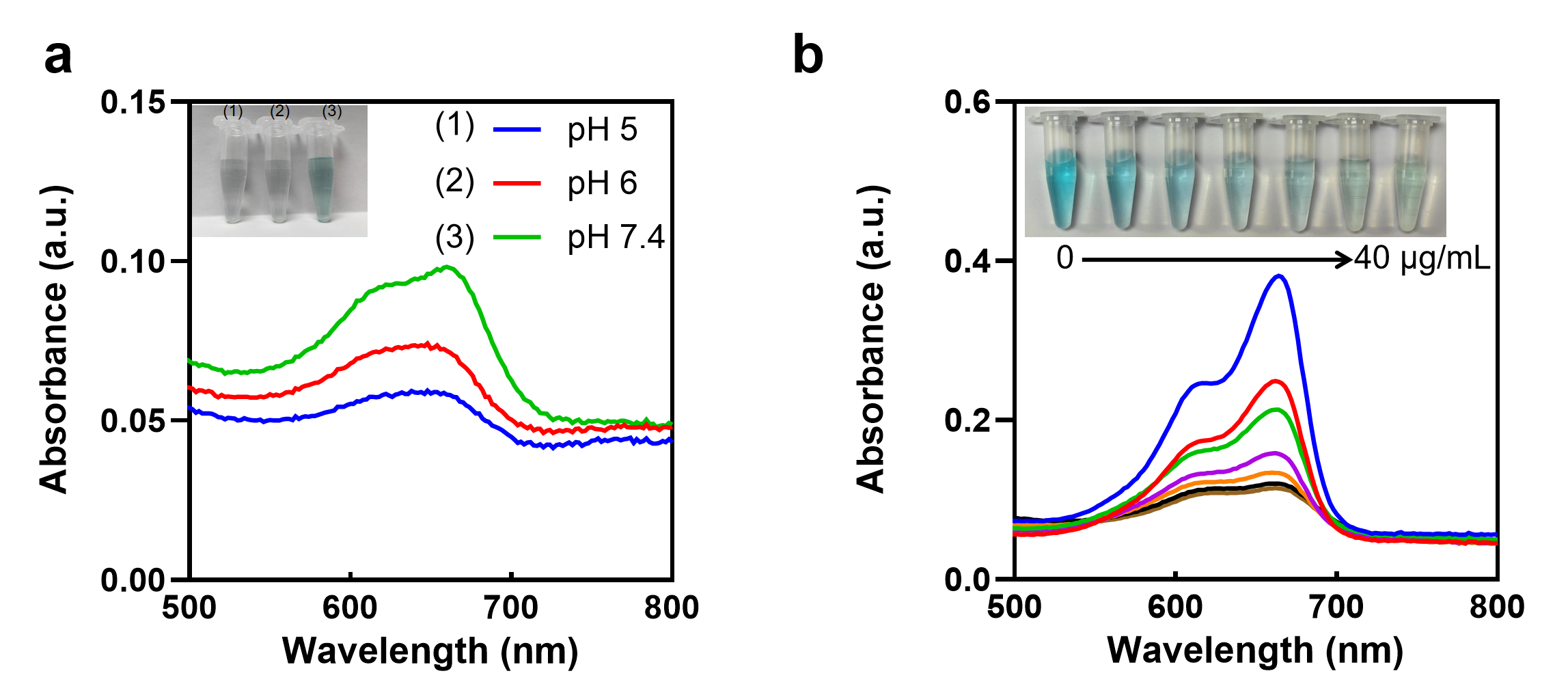


Figure S27. The UV–vis absorption spectra of the mixed solutions containing MB , H_2_O_2_ and Ti_3_C_2_-MnO_2_-PDA at (a) different pH levels and (b) different concentrations of Ti_3_C_2_-MnO_2_-PDA (0-40 μg/mLrespectively), and the color changes of the resulting solutions.


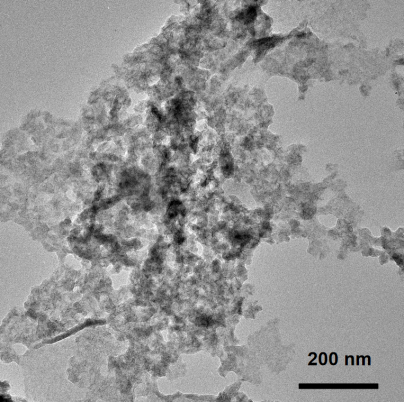


Figure S28. Representative TEM images of the nanoparticles at pH 6 Ti_3_C_2_-MnO_2_-PDA (200 nm).


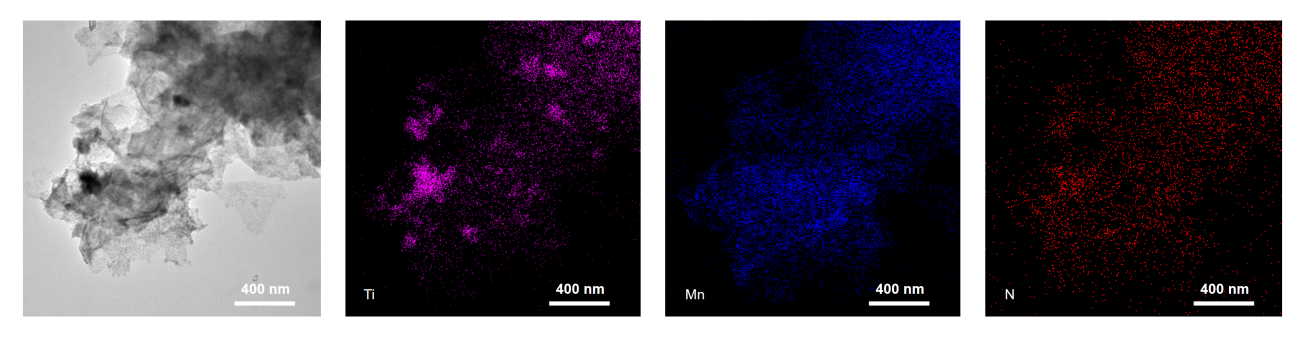


Figure S29. EDS elemental analysis proved Ti, Mn, and N of Ti_3_C_2_-MnO_2_-PDA nanoparticles for pH 7.4 (400 nm).


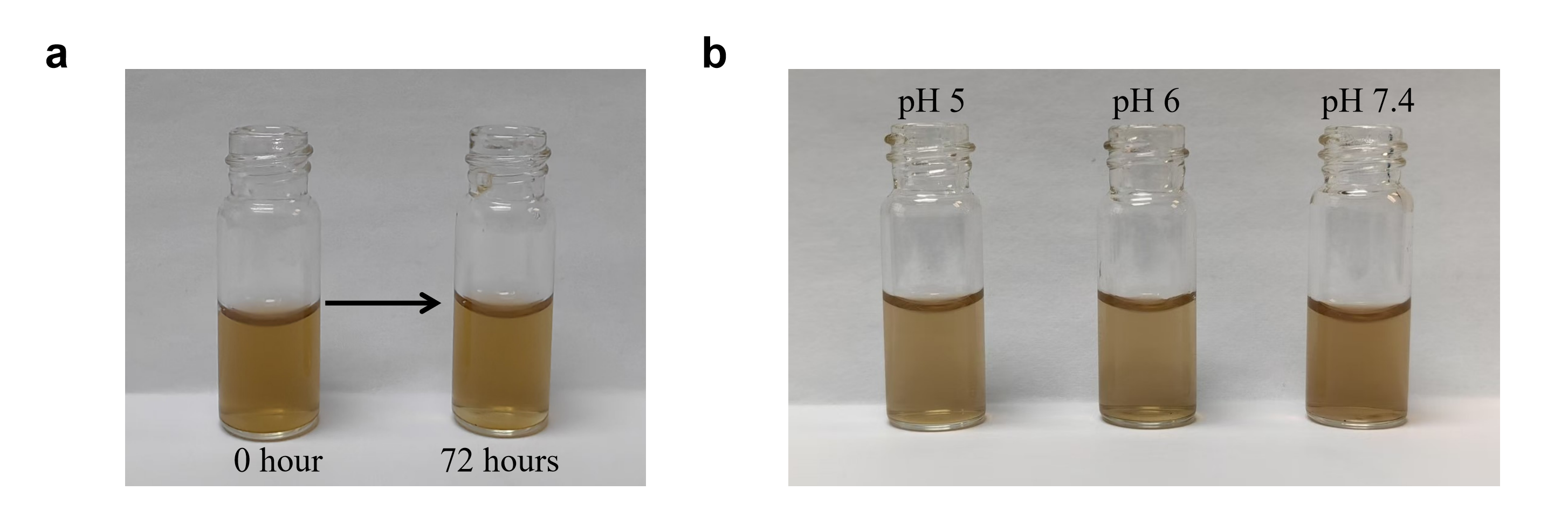


Figure S30. (a) Comparison chart of nanoparticles in serum for 72 hours with 0 hour and (b) nanoparticles in different pH media for 72 hours.


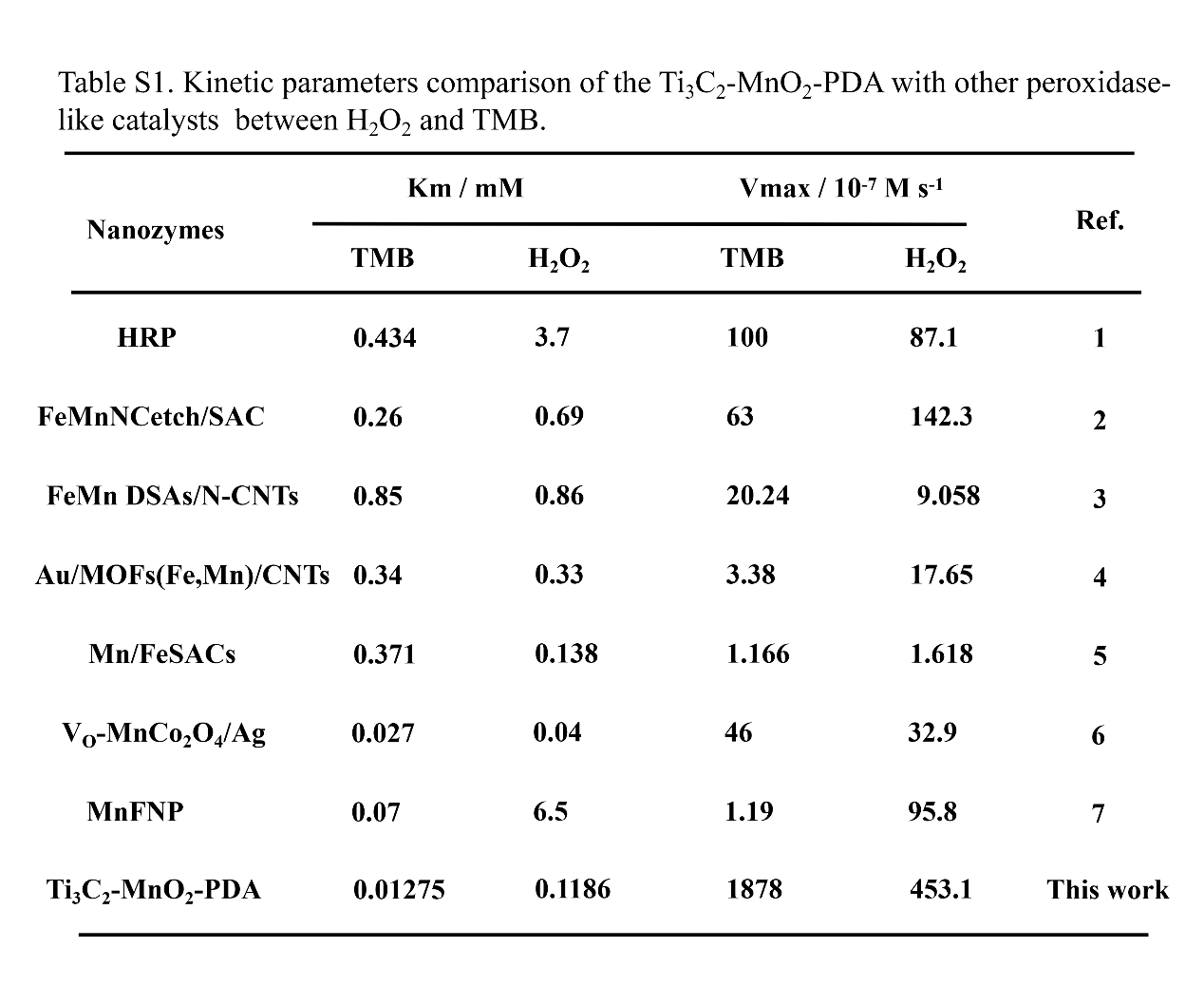
 Table S2. Antibodies were used for flow cytometry in this study

| Antibodies | FLUOROCHROME | CLONE | Company | PURPOSE |
| --- | --- | --- | --- | --- |
| Viability Staining Solution | 7-AAD |  | Biolegend | Viability Staining Solution |
| CD45 | Percp-cy5.5 | 30-F11 | Biolegend | Leukocytes |
| CD3 | AF700 | 17A2 | Biolegend | Pan T cells, NKT-Like cells |
| CD4 | APC-Fire810 | GK1.5 | Biolegend | CD4 T cells |
| CD8 | efluor450 | 53-6.7 | Biolegend | CD8 T cells |
| CD44 | efluor506 | IM7 | Biolegend | Flow |
| CD11b | pe/Dazzle594 | M1/70 | Biolegend | Flow |
| CD11c | PE | N418 | Biolegend | Flow |
| CD80 | APC | 16-10A1 | Biolegend | activated B and T cells, macrophages, DCs |
| CD86 | BV605 | GL1 | Biolegend | activated B and T cells, macrophages, DCs |
| CD40 | APC-CY7 | 3/23 | Biolegend | activated B and T cells, macrophages, DCs |
| MHCⅡ | FITC | M5/114.15.2 | Biolegend | Flow |

**REFERENCES**

[1] L. Gao, J. Zhuang, L. Nie, J. Zhang, Y. Zhang, N. Gu, T. Wang, J. Feng, D. Yang, S. Perrett, X. Yan, Intrinsic peroxidase-like activity of ferromagnetic nanoparticles, Nat Nanotechnol 2(9) (2007) 577-83.

[2] Y. Wang, R. Zeng, S. Tian, S. Chen, Z. Bi, D. Tang, D. Knopp, Bimetallic Single-Atom Nanozyme-Based Electrochemical-Photothermal Dual-Function Portable Immunoassay with Smartphone Imaging, Anal Chem 96(33) (2024) 13663-13671.

[3] Y.-W. Mao, J. Zhang, R. Zhang, J.-Q. Li, A.-J. Wang, X.-C. Zhou, J.-J. Feng, N-Doped Carbon Nanotubes Supported Fe–Mn Dual-Single-Atoms Nanozyme with Synergistically Enhanced Peroxidase Activity for Sensitive Colorimetric Detection of Acetylcholinesterase and Its Inhibitor, Analytical Chemistry 95(22) (2023) 8640-8648.

[4] X. Dang, H. Zhao, Bimetallic Fe/Mn metal-organic-frameworks and Au nanoparticles anchored carbon nanotubes as a peroxidase-like detection platform with increased active sites and enhanced electron transfer, Talanta 210 (2020) 120678.

[5] M. Zhang, W. Xu, Y. Gao, N. Zhou, W. Wang, Manganese–Iron Dual Single-Atom Catalyst with Enhanced Nanozyme Activity for Wound and Pustule Disinfection, ACS Applied Materials & Interfaces 15(36) (2023) 42227-42240.

[6] Y. Tan, M. Qi, H. Jiang, B. Wang, X. Zhang, Determination of uric acid in serum by SERS system based on VO-MnCo2O4/Ag nanozyme, Analytica Chimica Acta 1274 (2023) 341584.

[7] N.K. Dega, A.B. Ganganboina, H.L. Tran, E.P. Kuncoro, R.-a. Doong, BSA-stabilized manganese phosphate nanoflower with enhanced nanozyme activity for highly sensitive and rapid detection of glutathione, Talanta 237 (2022) 122957.
